# Molecular mechanisms underlying host-induced gene silencing

**DOI:** 10.1093/plcell/koac165

**Published:** 2022-06-06

**Authors:** Hana Zand Karimi, Roger W Innes

**Affiliations:** Department of Biology, Indiana University, Bloomington, Indiana 47405, USA; Department of Biology, Indiana University, Bloomington, Indiana 47405, USA

## Abstract

Host-induced gene silencing (HIGS) refers to the silencing of genes in pathogens and pests by expressing homologous double-stranded RNAs (dsRNA) or artificial microRNAs (amiRNAs) in the host plant. The discovery of such trans-kingdom RNA silencing has enabled the development of RNA interference-based approaches for controlling diverse crop pathogens and pests. Although HIGS is a promising strategy, the mechanisms by which these regulatory RNAs translocate from plants to pathogens, and how they induce gene silencing in pathogens, are poorly understood. This lack of understanding has led to large variability in the efficacy of various HIGS treatments. This variability is likely due to multiple factors, such as the ability of the target pathogen or pest to take up and/or process RNA from the host, the specific genes and target sequences selected in the pathogen or pest for silencing, and where, when, and how the dsRNAs or amiRNAs are produced and translocated. In this review, we summarize what is currently known about the molecular mechanisms underlying HIGS, identify key unanswered questions, and explore strategies for improving the efficacy and reproducibility of HIGS treatments in the control of crop diseases.

## Teaser

A review of what is known and unknown about the molecular mechanisms underlying the silencing of pathogen and pest genes via the expression of complementary RNAs in the host plant.

## Introduction

The phenomenon of RNA-induced gene silencing (also known as RNA interference [RNAi]) was first described in plants over 30 years ago, when plant scientists attempted to overexpress a gene involved in purple pigment production in petunia (*CHALCONE SYNTHASE1*), but instead silenced this gene through a posttranscriptional mechanism (Napoli et al., 1990). A similar phenomenon was also described in fungi during this time period and was named “quelling” (Romano and Macino, 1992; Cogoni and Macino, 1997). Quelling was originally discovered in the fungus *Neurospora crassa* when the expression of either the *albino-1* or *albino-3* gene resulted in silencing of endogenous *albino-1* or *albino-3*. Although the mechanism of quelling was not clear at the time, the silencing of *albino* genes was correlated with reductions in mRNA levels. Later studies on quelling-defective (QDE) mutants showed that the corresponding genes encoded core proteins/enzymes that are now known to mediate RNAi across eukaryotes: RNA-dependent RNA polymerase (QDE-1; Cogoni and Macino, 1999) and Argonaute (AGO) proteins (QDE-2; Fagard et al., 2000).

RNA-induced gene silencing in eukaryotes is mediated by small RNAs (sRNAs; 20–24 nt long) that associate with AGO proteins and then base-pair with complementary mRNAs. The AGO proteins can then slice the mRNA, inhibit translation of the mRNA, or recruit protein complexes that mediate RNA-directed DNA methylation of complementary DNA (reviewed in Carbonell, 2017). The specificity of such gene silencing complexes is dictated by the sRNAs, which are typically produced from longer self-complementary hairpin RNAs (hpRNAs) or double-stranded RNAs (dsRNAs; produced using flanking inverted promoters) by dsRNA-cleaving enzymes belonging to the Dicer family (DICER-LIKE [DCL] proteins). This RNAi system is highly conserved among eukaryotes, with functional AGO and DCL proteins known to be expressed in nematodes, insects, fungi, oomycetes, plants, and mammals, including humans (Hammond et al., 2000; Bernstein et al., 2001; Vetukuri et al., 2011; Campo et al., 2016).

In plants, transgenes can be silenced by both transcriptional and posttranscriptional mechanisms. Transcriptional gene silencing (TGS) is associated with the DNA methylation of the target gene, which inhibits the binding of RNA polymerase II (Pol II; Rountree and Selker, 1997; Portela and Esteller, 2010). Post-TGS (PTGS) is a consequence of mRNA degradation or the inhibition of translation (Eamens et al., 2008; Guo et al., 2016). The overexpression of sense or antisense gene constructs can induce homologous gene silencing; however, the expression of dsRNAs or hpRNAs is much more effective in silencing target genes (Waterhouse et al., 1998; Wang and Waterhouse, 2000), with optimal silencing achieved by the inclusion of an intron in the hpRNA construct (Smith et al., 2000; Wesley et al., 2001). These studies were key breakthroughs that led to the wide use of RNAi as a tool for the analysis of gene function in plants (Waterhouse and Helliwell, 2003).

The first evidence that silencing-related RNAs produced in plants might be able to silence genes in another organism came from work done with the free-living nematode *Caenorhabditis elegans* (Boutla et al., 2002). In that work, total RNA was purified from transgenic *Nicotiana benthamiana* plants expressing a gene encoding green fluorescent protein (*GFP*) that had been silenced. RNA gel-blot analyses of this total RNA showed that it included siRNAs complementary to both the sense and anti-sense strands of *GFP*. Injection of this total RNA into transgenic *C. elegans* resulted in silencing of *GFP*, showing that the silencing activity produced by the plant was functional in the nematode. Notably, size fractionation of the RNA showed that the most active fraction was 80-90 nt in length, which suggested that the silencing activity was conferred by longer dsRNAs rather than mature siRNAs.

One of the first applications of RNAi to study plant pests or pathogens involved soaking nonfeeding juvenile (J2) stage soybean cyst nematodes (*Heterodera glycines*) in a buffered solution containing dsRNA, which was found to be taken up by an oral route (Urwin et al., 2002). Bakhetia et al. (2005) used a similar juvenile feeding approach to silence peroxidase and NADPH oxidase genes in the root knot nematode *Meloidogyne incognita*. They observed phenotypic changes in nematode development up to 35 days after exposure to dsRNA, and fewer nematodes and eggs were recovered from plants infected with dsRNA-treated versus control-treated nematodes (Bakhetia et al., 2005). These results suggested that transgenic expression of dsRNA targeting nematode virulence genes might be an effective approach to suppressing infection by nematodes, and potentially other plant pests and pathogens.

This hypothesis was confirmed in 2006, when Huang and colleagues reported, for the first time, host-induced gene silencing (HIGS) of a nematode gene (Huang et al., 2006). The authors expressed hpRNA in *Arabidopsis thaliana* that targeted the parasitism gene 16D10, which encodes a peptide secreted into the saliva of root-knot nematodes. Transgenic Arabidopsis expressing 16D10 hpRNA exhibited effective resistance against four major root knot nematode species, highlighting the promise of using HIGS to engineer disease-resistant crops (Huang et al., 2006; Lilley et al., 2007). Numerous studies soon followed that employed HIGS in crop plants to confer resistance to diverse plant pathogens and pests, including fungi (Nowara et al., 2010; Koch et al., 2013), oomycetes (Govindarajulu et al., 2015; Jahan et al., 2015), and insects (Abdellatef et al., 2015; Zhang et al., 2015b). For example, HIGS has been used in many different crops to target fungal pathogen genes, including barley (*Hordeum vulgare:* targeting genes in *Blumeria graminis* and *Fusarium graminearum*; Nowara et al., 2010; Koch et al., 2013), wheat (*Puccinia triticina* and *F. graminearum*; Nowara et al., 2010; Panwar et al., 2013), soybean (*Fusarium*  *oxysporum*; Kong et al., 2022), maize (*Aspergillus flavus*; Raruang et al., 2020), and banana (*F. oxysporum*; Ghag et al., 2014). HIGS has also been used against insect pests including aphids (Guo et al., 2014), Colorado potato beetles (Zhang et al., 2015b), and cotton bollworm (Mamta et al., 2016).

Although HIGS represents a promising approach for limiting crop losses caused by pathogens and pests, many questions remain, including how silencing RNAs are secreted from plant cells, and how cells in the pathogen or pest take up these RNAs. It is also not clear which proteins from the plant and pathogen are required for successful HIGS. In this review, we focus on the mechanisms underlying HIGS. We also discuss recent findings highlighting how plants use endogenous RNAi to overcome pathogen infection, how plant cells recognize long dsRNA and produce small interfering RNAs (siRNAs), and how we can improve the efficacy of siRNA production using various dsRNA constructs. We describe possible mechanisms of RNA delivery, including whether extracellular vesicles (EVs) play a role in this process. Lastly, we suggest future directions aimed at addressing the major unanswered questions about how HIGS functions and how it can be improved.

## Molecular mechanisms underlying HIGS

The molecular mechanisms underlying HIGS may differ between insects and nematodes versus fungi and oomycetes (filamentous pathogens). For the herbivorous insects, it appears that long dsRNAs (including hpRNAs) are taken up directly from the host and then processed using the RNAi machinery within the insect or nematode to induce gene silencing. For filamentous pathogens, the available evidence suggests that siRNAs and microRNAs (miRNAs) produced within the host plant are taken up by the pathogen, which then induce gene silencing, although in most systems, translocation of long dsRNA has not been ruled out. Here we discuss key experiments addressing these issues within each pest and pathogen group.

### Long dsRNAs and sRNAs both induce gene silencing in insects

In herbivorous insects, the translocation of dsRNA appears to be more effective at inducing gene silencing than the translocation of siRNAs. This was demonstrated by comparing the HIGS efficacy of dsRNA expression inside chloroplasts versus the nucleus. Chloroplast-expressed dsRNAs are protected against host cell DCL enzymes and thus can accumulate to much higher levels than dsRNAs expressed from nucleus-encoded genes, which are rapidly processed into siRNAs (Zhang et al., 2015b; Bally et al., 2016). Importantly, the efficacy of HIGS against two herbivorous insect species, Colorado potato beetle (*Leptinotarsa decemlineata*) and cotton bollworm (*Helicoverpa armigera*), was much higher when dsRNA was expressed inside chloroplasts, indicating that long dsRNA is the key translocated RNA, rather than siRNAs. Such a plastid-based system is unlikely to work against phloem-feeding insects, however, as these insects are unlikely to ingest whole chloroplasts.

The conclusion that insect’s RNAi machinery contributes to efficient HIGS in insects is further supported by a recent study from Bally et al. (2020) in which gene silencing was induced in *H. armigera* by expressing an artificial miRNA (amiRNA) construct in *N. benthamiana* that used the backbone of an insect miRNA precursor gene. This modified amiRNA precursor remained largely unprocessed in *N. benthamiana* due to the lack of recognition by plant DCLs. However, feeding on transgenic leaves expressing this construct led to efficient silencing of *H. armigera* genes and high levels of mortality and growth abnormalities. These results indicate that the unmodified precursor of the amiRNA was taken up by insect cells and processed by the insect’s RNAi machinery.

Although long dsRNAs and hpRNAs can clearly be taken up by insects, miRNAs and siRNAs can also induce silencing in insects. For example, amiRNAs based on plant miRNA precursors and siRNAs derived from the expression of long dsRNAs silence genes in aphids (Pitino et al., 2011; Guo et al., 2014; Abdellatef et al., 2015). Remarkably, in one study, silencing appeared to persist through multiple parthenogenetic generations, even after the aphids (*Sitobion avenae*) were switched back to feeding on nontransgenic plants (Abdellatef et al., 2015). These results suggest that HIGS can induce epigenetic changes in aphids that are heritable, or perhaps that aphids produce secondary siRNAs that can be passed onto subsequent asexual generations.

Once dsRNAs are consumed by insects, they must somehow cross the plasma membrane of intestinal epithelial cells and engage with host cell RNAi machinery. One potential route of uptake is endocytosis. Xiao et al. (2015) showed that inhibiting clathrin-dependent endocytosis significantly reduced the uptake of dsRNA and impaired RNAi in red flour beetles (*Tribolium castaneum*). Consistent with this finding, silencing of the gene encoding the clathrin heavy chain in Colorado potato beetles reduced target gene silencing, as did pharmacological inhibition of endocytosis (Cappelle et al., 2016). In this latter study, however, co-silencing of two putative dsRNA transporter genes (*Systemic Interference Defective* 1 (*sid-1*)-like A and C (*SilA* and *SilC*)) also reduced target gene silencing, suggesting that dsRNA may also be taken up via a channel-based mechanism. The *sid-1* gene was first identified in the nematode *Caenorhabditis elegans* when screening for mutants lacking the systemic RNAi phenotype (Feinberg and Hunter, 2003), and the Sid-1 protein was then shown to mediate dsRNA import into cells (Winston et al., 2002). *Sid-1*-like genes were subsequently identified in multiple insect species, including cotton/melon aphid (*Aphis gossypii*; Xu and Han, 2008), grasshopper (*Schistocerca americana*; Dong and Friedrich, 2005), honeybee (*Apis mellifera*; Honeybee Genome Sequencing, 2006), and planthopper (*Nilaparvata lugens*; Xu et al., 2013). However, the work of Capelle et al. is the first to show a functional link between Sid-1 and HIGS in insects.

### Long dsRNAs and sRNAs also induce gene silencing in filamentous pathogens

How silencing RNAs are taken up by filamentous pathogens is also poorly understood, but recent studies have demonstrated that many species can take up naked dsRNA directly from the environment (Wang et al., 2016; Qiao et al., 2021). The RNA uptake efficiency of six fungal pathogens and the oomycete plant pathogen *Phytophthora infestans* was assessed using fluorescein-labeled dsRNA. Notably, dsRNA uptake efficiency varied among species and correlated with the efficiency of gene silencing mediated by exogenous RNA in each species. This finding suggests that HIGS in at least some filamentous pathogens can be mediated by the direct uptake of naked dsRNAs, as opposed to siRNAs produced by the plant or dsRNAs packaged by the plant. This hypothesis is further supported by the finding that mutating the *DCL1* gene in the necrotrophic fungus *F. graminearum* rendered it insensitive to gene silencing when dsRNA was sprayed on detached barley leaves but had no effect on its sensitivity to silencing when siRNAs were used (Koch et al., 2016). These results demonstrate that dsRNAs are taken up by fungal plant pathogens and can then engage with the fungal RNAi machinery to silence fungal genes. That said, it is also clear that fungal pathogens can take up siRNAs directly from the environment and that these siRNAs can also silence target genes (Wang et al., 2016), bypassing the requirement for fungal DCL proteins (Koch et al., 2016). Thus, for transgenic plants expressing nucleus-encoded hpRNA constructs, it is possible that both dsRNAs and siRNAs contribute to the silencing of pathogen genes, with the relative contribution of each depending on the efficiency at which hpRNAs are processed into siRNAs by plant DCLs and the efficiencies at which hpRNAs and siRNAs are secreted.

### Optimizing artificial sRNA production in plants

**Figure 1:**
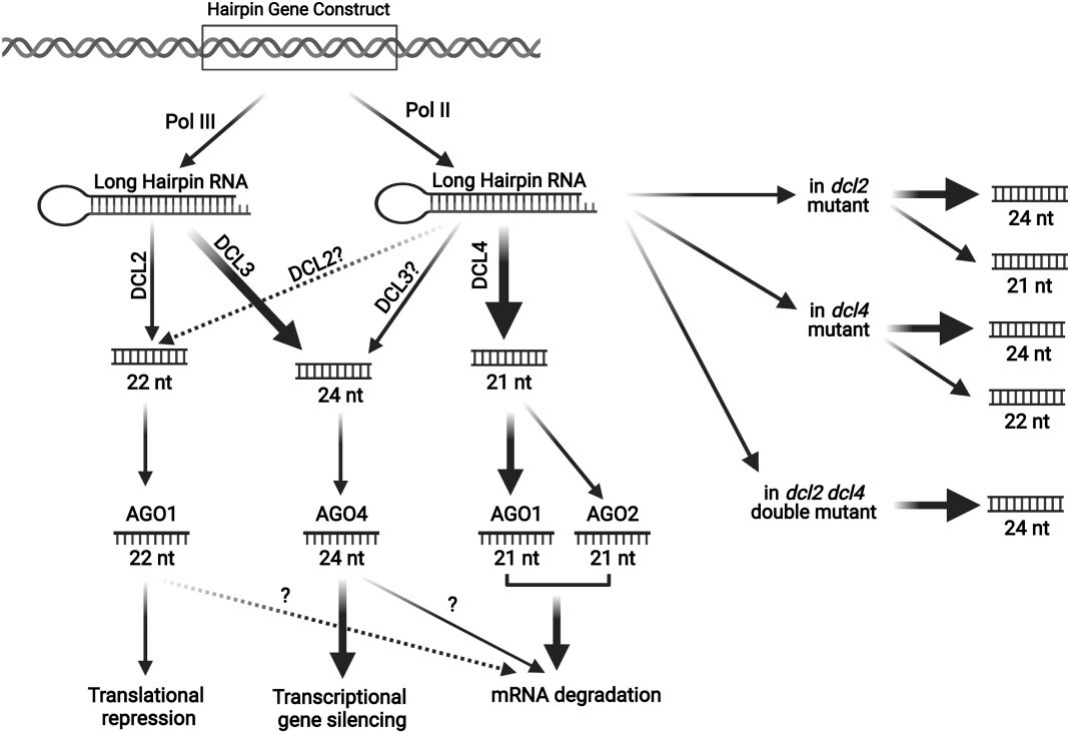
Pathways for the production of siRNAs in Arabidopsis. hpRNA constructs can be expressed using either Pol II or Pol III promoters (Wang et al., 2008). hpRNAs synthesized by RNA Pol III localize to the nucleolus and are mostly targeted by DCL3 and slightly by DCL2, resulting in the production of mostly 24-nt sRNAs and some 22-nt sRNAs (Wang et al., 2008). hpRNAs synthesized by RNA Pol II are primarily targeted by DCL4, predominantly producing 21-nt sRNAs, with a small contribution of DCL2 and DCL3 in the absence of DCL4 function (Fusaro et al., 2006). AGO1 and AGO2 preferentially bind to 21-nt sRNAs and can then cleave homologous mRNAs. AGO1 can also bind to 22-nt sRNAs when the latter are at high concentrations and can then inhibit the translation of target mRNAs (Baumberger and Baulcombe, 2005; Wu et al., 2020). Whether AGO1-bound 22-nt sRNAs are able to induce mRNA degradation is not yet clear. The 24-nt sRNAs mainly bind to AGO4 and direct TGS via DNA methylation (Zilberman et al., 2004).

The processing of long hpRNAs into siRNAs in plants is primarily mediated by DCL2, 3, and 4, which collectively produce 21-, 22-, and 24-nt siRNAs (Figure 1; Fusaro et al., 2006). DCL3 is specifically required for the production of 24-nt siRNAs, and DCL4 is required for the production of 21-nt siRNAs, while DCL2 contributes to the production of 22-nt siRNAs, but only in the absence of DCL4 (Fusaro et al., 2006). In an Arabidopsis *dcl2 dcl4* double mutant background, production of 21- and 22-nt siRNAs derived from the hpRNA is largely eliminated, as is the degradation of target mRNAs within the plant.

In contrast, the elimination of DCL3, which blocks the production of 24-nt siRNAs, enhances PTGS. Although paradoxical, this can be explained by an increase in transcription of the hpRNA due to elimination of RNA-directed DNA methylation of the promoter driving hpRNA expression. This increase in hpRNA production should then increase 21- to 22-nt siRNA production, and thus lead to more efficient degradation of the target mRNA (Fusaro et al., 2006). Thus, 21- and 22-nt siRNAs appear to be the primary mediators of PTGS within a plant. Whether this is also true for gene silencing in plant pathogens is not yet clear. However, 21 and 22-nt sRNAs mediate RNAi in insects such as the fruit fly *Drosophila melanogaster* (Elbashir et al., 2001).

As mentioned above, amiRNAs have also been successfully used in transgenic plants to silence genes in insects (aphids) and filamentous pathogens. Optimizing the expression of such amiRNAs is thus one avenue toward improving the efficacy of HIGS. Standard amiRNA constructs are based on endogenous primary miRNA (pri-miRNA) genes, which encode RNAs that form complex hairpin structures that are processed by DCL1 in plants to produce 21- or 22-nt mature miRNAs. amiRNAs are generated by replacing just the helical region of the pri-miRNA corresponding to the mature miRNA. Optimizing amiRNA production thus involves optimizing the expression and processing of the pri-amiRNA. For example, the loop region in pri-miRNAs plays an important role in controlling the maturation of pri-miRNAs and can directly contribute to the repression of target mRNAs, at least in human cells (Yue et al., 2011). In addition, for some human pri-miRNAs, the loop sequences can also be processed into functional miRNAs. Indeed, in some miRNA libraries, the read count for loop-miRNAs is higher than that of the corresponding mature miRNAs, suggesting they can be functional, rather than just being the byproducts of pri-mRNA processing (Winter et al., 2013). Consistent with this notion, loop-miRNAs bind to AGO2 protein in human cells and induce the degradation of matching mRNAs (Winter et al., 2013). These observations suggest that in some cases, the loop regions of pri-miRNAs might trigger gene silencing. Thus, one can envision designing amiRNAs that produce two distinct miRNAs, potentially targeting two different transcripts or two different positions in a single transcript. Whether this is also true for plant pri-miRNAs is not known, but the modification of pri-amiRNA loop sequences might offer one avenue for optimizing amiRNA efficacy.

Because pri-amiRNAs are processed by DCL1, they should not lead to the production of 24-nt siRNAs. Therefore, amiRNA-encoding genes should be less prone to TGS via RNA-directed DNA methylation, which is a known problem for hpRNA constructs (Zilberman et al., 2004). However, a study in wheat revealed a higher frequency of silencing of an amiRNA construct in the T2 generation relative to a hpRNA construct targeting the same gene (Gasparis et al., 2016); thus, this expectation requires further assessment. A second potential advantage of amiRNAs versus hpRNAs is that amiRNAs should be less likely to have off-target effects given that they produce a single amiRNA, rather than a family of siRNAs with multiple different sequences. Notably, a direct comparison of dsRNA and amiRNA constructs, both expressed in tobacco (*Nicotiana tabacum*) plants under the control of the CaMV 35S promoter, showed similar efficacies in reducing the fecundity of the aphid *Myzus persicae* (Guo et al., 2014). Both constructs targeted the aphid gene *Acetylcholinesterase2*, but only the amiRNA construct triggered a statistically significant reduction in mRNA levels of the target gene (Guo et al., 2014). This is a single study with single species, however. Similar comparisons are lacking for oomycete and fungal pathogens.

Loop structures in hpRNAs are also known to affect silencing efficacy. Both loop size and loop sequence can affect hpRNA stability (Groebe and Uhlenbeck, 1988; Antao and Tinoco, 1992; Serra et al., 1993; Vecenie and Serra, 2004; Kuznetsov et al., 2008), which consequently can affect siRNA production. hpRNAs with shorter loops are generally more stable than hpRNAs with longer loops (Groebe and Uhlenbeck, 1988). The stem sequence also affects the stability of hpRNAs, with higher GC content producing more stable hairpins (Groebe and Uhlenbeck, 1988). Thus, the optimization of hpRNA constructs to maximize HIGS may involve optimizing the length of the loop and the sequences within the stem.

The entire stem region of hpRNAs likely contributes to siRNA production, but the length of the hpRNA stem is not critical to efficacy, with stem lengths ranging from 98 to 730 nt all shown to be efficient in triggering target gene silencing (Wesley et al., 2001). More critical to hairpin design is the inclusion of an intron in the construct, which is typically placed at the position of the loop, although an intron located 5′ of the stem structure also appeared to promote silencing (Wesley et al., 2001). Why introns promote target gene silencing is not yet understood but presumably results from more efficient siRNA production. This could be related to the recruitment of spliceosome RNA–protein complexes that somehow promote further processing by DCL proteins.

### Optimizing target gene selection for HIGS

The efficacy of HIGS also depends on the genes selected for silencing in the pathogen. Some genes are not suitable as targets for HIGS, because their knockdown does not affect pathogenesis (Govindarajulu et al., 2015). Singh et al. (2018) demonstrated that housekeeping genes can make effective targets for HIGS, at least in insects. The authors targeted housekeeping genes in cotton leafhopper via feeding dsRNAs and observed up to 48% mortality (Singh et al., 2018). A caveat of this approach, however, is that housekeeping genes are usually highly conserved between organisms; thus, targeting these genes could be detrimental to nontarget organisms, and potentially even to the host plant. An ideal target gene would be one that is essential for viability of the pathogen/pest on the host plant but is not found in related beneficial organisms or the host plant.

A second concern when designing HIGS constructs is the potential for the resulting siRNAs to silence off-target genes (Xu et al., 2006; Senthil-Kumar and Mysore, 2011). It is estimated that 50%–70% of gene transcripts in plants have the potential to generate siRNAs that either target more than one gene or target endogenous plant genes (Xu et al., 2006). To minimize this risk, computational approaches have been developed to select a collection of 21-nt sequences from a given target sequence with a low probability of such off-target effects, while still being efficiently loaded into AGO proteins (Ahmed et al., 2020). This approach was validated in *N. benthamiana* using virus-induced gene silencing (VIGS) targeting two different genes, the phytoene desaturase gene and the ribosomal protein L10 (*RPL10*) gene. RNA-seq analysis of total transcripts in *N. benthamiana* following infection with VIGS constructs designed to either maximize or minimize off-target silencing confirmed that the tool was effective at minimizing off-target effects while maximizing target gene silencing (Ahmed et al., 2020).

In the context of HIGS, the efficacy and specificity of candidate siRNA sequences can often be tested on pathogens grown in Petri dishes or other artificial environments prior to the generation of transgenic plants. For example, Guo et al. (2019) tested the effect of artificial siRNAs (asiRNAs) that targeted several different genes in the rice fungal pathogen *M. oryzae*, including *MoAP1*, which encodes a transcription factor essential for conidia production. Application of asiRNAs targeting *MoAP1* to *M. oryzae* in a Petri dish inhibited the growth of the pathogen and suppressed pathogenicity on rice (Guo et al., 2019). In contrast, feeding *M. oryzae* asiRNAs that targeted three genes downstream of *MoAP1* failed to affect fungal growth or pathogenicity, revealing that these latter three genes were likely not good targets for HIGS. Based on these analyses, the authors expressed an hpRNA construct in transgenic rice that targeted *MoAP1* and observed enhanced resistance to multiple strains of *M. oryzae*. Thus, direct RNA applications/feeding can be useful for evaluating candidate target genes for HIGS.

Genes that function at an early stage of the infection process appear to be particularly good targets for HIGS. For example, *MoAP1* is required for the formation of appressoria, which are required by *M. oryzae* for the penetration of host cell walls (Guo et al., 2019). Similar observations were made for the fungal pathogens *P.*  *triticina* (Panwar et al., 2013) and *Puccinia striiformis* f. sp. *tritici* (Zhu et al., 2017; Qi et al., 2018), in which genes highly expressed in early stages of infection were targeted by HIGS, resulting in robust resistance. These observations suggest that the siRNAs are transferred into fungi prior to the formation of haustoria, possibly from the leaf surface during the growth of germ tubes. The recent discovery that plants secrete RNA into the leaf apoplast (Zand Karimi et al., 2022) raises the possibility that RNA can be deposited onto the leaf surface.

Although the above approaches should enable the selection of target genes and optimal target sequences, it should be noted that the process of siRNA biogenesis in plants often leads to the spread of siRNA production along a target mRNA. For example, gene silencing can initiate in the 3′- untranslated region (UTR) of a target gene and spread to the coding regions (English et al., 1996; Sijen et al., 2001). As a classic example of this, constructs consisting of a single copy of a target gene fused to an inverted repeat of the 3′-UTR region of the nopaline synthase gene from *Agrobacterium tumefaciens* are highly efficient at silencing target genes in transgenic plants, even though the target genes have a completely different 3′-UTR (English et al., 1996; Sijen et al., 2001). As another example, when a transgenic Arabidopsis plant expressed a full-length green fluorescent protein (GFP) construct, hpRNAs targeted the 5′ half of the *GFP* gene led to the production of secondary siRNAs from the 3′ half (Taochy et al., 2017). Such spreading is dependent on the host’s RNA-DEPENDENT RNA POLYMERASE 6 (RDR6; Taochy et al., 2017), indicating that RDR6 plays a central role in the production of secondary siRNAs. These observations also point to the potential for off-target effects resulting from secondary siRNA biogenesis. This may be less of a concern for HIGS constructs if the target gene in the pathogen or pest has no similarity to endogenous plant genes.

### Promoter selection for HIGS constructs can affect both the efficacy and specificity of HIGS

In addition to selecting the appropriate pathogen gene and target site sequence within that gene, HIGS efficacy is likely dependent on obtaining high levels of hpRNA and/or siRNAs in the tissue/cells colonized by the pathogen or pest. Most HIGS experiments have employed strong constitutive promoters to accomplish this goal, such as the 35S promoter of cauliflower mosaic virus, which is transcribed by RNA Pol II. An RNA Pol III promoter could also be used to drive high expression of hpRNAs, but such promoters have proven to be less effective in HIGS (Wang et al., 2008). This is likely because Pol II-derived hairpins are mostly processed by DCL4, resulting in the production of 21-nt siRNAs, whereas Pol III-derived siRNAs are processed by DCL3 to produce mostly 24-nt siRNAs (Figure 1; Wang et al., 2008). Since 24-nt siRNAs are known to mediate TGS through DNA methylation, hpRNA constructs transcribed by Pol III are more likely to be silenced and are thus not a good choice for sustained HIGS (Gasciolli et al., 2005; Fusaro et al., 2006; Wang et al., 2008).

Another consideration when designing HIGS constructs is whether to use constructs that generate dsRNAs using flanking inverted promoters to drive the expression of sense and anti-sense strands from the same insert or constructs that generate hpRNAs expressed from a single promoter. A potential problem with the former approach is a higher propensity for transcriptional silencing of the HIGS construct due to the production of RNA from the promoter regions. Nevertheless, such inverted promoter constructs have been used to silence target genes in *F. graminearum* in both barley and Arabidopsis (Koch et al., 2013), although only T2 generations were tested.

An alternative to strong constitutive promoters is to select promoters that drive the expression of hpRNAs specifically in the tissue and cell types being attacked. Alakonya et al. (2012) provided a nice example of this approach, in which they used HIGS to engineer tobacco with resistance to colonization by the parasitic plant *Cuscuta pentagona* (dodder). Most parasitic plants establish vascular connections with their host plants via structures called haustoria. Alakonya et al. (2012) used a phloem-specific promoter from the sucrose transporter gene *SUC2* in Arabidopsis to drive expression of a hpRNA targeting the *STM* gene of dodder, which is required for haustoria formation. Phloem-specific expression of this hpRNA was highly effective at reducing dodder growth on tobacco. The use of a tissue-specific promoter in this example localizes expression of the hpRNA to areas of likely infection while limiting expression in other parts of the plant, which should reduce potential off-target effects on host genes. Given that HIGS appears to act very early during the fungal infection process, prior to the formation of haustoria (Guo et al., 2019), the use of pathogen-inducible promoters may not be optimal, as siRNA production might occur too late to be effective.

### HIGS likely requires AGO proteins, but whether they are supplied by the host or pathogen is not yet known

To silence target genes, siRNAs and miRNAs must associate with AGO proteins. AGO proteins bind the dsRNA products of DCLs and slice and eject one of the strands, leaving a single-stranded guide RNA. Plant genomes typically contain multiple *AGO* genes. For example, the Arabidopsis genome (a dicot) contains 10 AGO genes (Zhang et al., 2015a), and the *Brachypodium* genome (a monocot) contains 16 (Secic et al., 2019). Plant AGO genes cluster into three major clades. In Arabidopsis, these include AGO1/5/10 (clade I), AGO2/3/7 (clade II), and AGO4/6/8/9 (clade III; Zhang et al., 2015a).

Individual AGO proteins differ in their binding preferences for sRNAs, especially with regards to preferred 5′-nucleotides and preferred lengths (Zhang et al., 2015a). They also differ in their expression patterns and responses to biotic and abiotic stress, as well as their mechanism of action (Zhang et al., 2015a; Figure 1). Clade 1 AGOs have primarily been implicated in binding 21-nt miRNAs and function in regulating developmental pathways via posttranscriptional mechanisms (Baumberger and Baulcombe, 2005; Wu et al., 2009). AGO2 (clade II) binds to both 21-nt miRNAs and 21-nt siRNAs and plays a central role in anti-viral and anti-bacterial immune responses (Zhang et al., 2011; Brosseau and Moffett, 2015). In contrast, AGO4 (clade III) binds to 24-nt siRNAs and functions in RNA-directed DNA methylation to epigenetically silence the transcription of transposable elements (Havecker et al., 2010). Notably, AGO3 (a clade II member derived from a tandem duplication of *AGO2*) also preferentially binds 24-nt RNAs but appears to function by inhibiting the translation of targeted mRNAs, as it associates with polysomes in the cytoplasm (Jullien et al., 2020). However, *AGO3* also partially complemented the *ago4* mutant when driven by the *AGO4* promoter, suggesting that it also functions in RNA-directed DNA methylation (Zhang et al., 2016b).

It is not yet clear whether plant siRNAs bind to pathogen AGO proteins, or if siRNAs are translocated as plant AGO-siRNA complexes. It is also not yet clear whether silencing in pathogens is achieved primarily through a posttranscriptional mechanism (e.g. cleavage of pathogen mRNAs) or through TGS (e.g. DNA methylation).

To understand the function of plant-derived sRNAs in pathogens, it is necessary to investigate the RNAi pathway in pathogens, and in particular, to assess whether DCL and/or AGO proteins in the pathogen are required for HIGS. In some fungal pathogens such as *Mucor circinelloides*, two classes of 21 and ∼25-nt siRNAs are produced from the expression of extra-chromosomal DNA. These two classes of siRNAs cause PTGS of target genes in *M. circinelloides*, indicating that this fungus possesses all the machinery required to carry out RNAi (Nicolas et al., 2003). Similarly, in the fungus *M. oryzae*, expression of hpRNAs results in the production of three different sizes of siRNAs ranging from ∼19 to ∼23 nt containing both sense and antisense strands (Kadotani et al., 2003). Surprisingly, in *M. oryzae*, all siRNA size classes are generated by just one of the two existing DCL proteins (Kadotani et al., 2004). Although siRNA production in fungi and plants looks similar, it is not known what size siRNAs are responsible for transcriptional or PTGS in fungi. As mentioned above, fungi can take up free dsRNAs from their environment, leading to gene silencing, and thus they must be employing their own DCLs and AGOs, or functional equivalents. Nguyen et al. (2018) investigated the function of three AGO proteins in *M. oryzae*, MoAGO1, MoAGO2, and MoAGO3, and showed that, although knockout mutations in *AGO1* and *AGO3* reduced the levels of gene silencing, the deletion of *MoAGO2* resulted in higher efficiency of gene silencing when triggered by hpRNAs expressed in the fungus. These observations confirm the notion that fungal AGOs function in gene silencing, and they indicate that different fungal AGOs perform different functions, just as observed in plants and mammals.

Fungal DCL proteins have also been shown to function in gene silencing. Knockout of *DCL1* and *DCL2* in the fungal plant pathogen *Colletotrichum higginsianum* derepressed a dsRNA mycovirus, and immunoprecipitation of sRNAs associated with fungal AGO1 showed abundant loading of viral siRNA. These findings indicate that the RNAi machinery in fungi plays an important role in viral defense, similar to its role in plants (Campo et al., 2016). It is thus tempting to speculate that HIGS of fungal pathogens is mediated by the fungal RNAi machinery, rather than by the transfer of plant AGO–siRNA complexes, but this remains to be tested.

### Pathogen-derived sRNAs are transferred to host cells and contribute to virulence

The presence of AGO and DICER-LIKE proteins in fungal and oomycete pathogens raises the possibility that sRNAs from these pathogens contribute to virulence. In other words, trans-kingdom RNA-mediated silencing may be bidirectional. The first report documenting the transfer of fungal sRNAs into host cells focused on the necrotrophic pathogen *Botrytis cinerea* during Arabidopsis infection (Weiberg et al., 2013). Evidence for transfer included immunoprecipitation of Arabidopsis AGO1 followed by reverse transcription–PCR. Three different fungal sRNAs associated with Arabidopsis AGO1, but not with Arabidopsis AGO2 or AGO4. In addition, *N. benthamiana* transiently expressing a putative target gene fused to GFP showed reduced GFP accumulation when infected by *B. cinerea*, but not when the target site of the sRNA was mutated. Lastly, overexpression of the *B. cinerea* sRNAs in Arabidopsis using an amiRNA construct rendered Arabidopsis more susceptible to *B. cinerea.* Together, these findings support a role for trans-kingdom RNA silencing in the virulence of *B. cinerea.*

Further support for pathogen-induced gene silencing in hosts comes from recent work with the oomycete *Hyaloperonospora arabidopsidis*, which infects Arabidopsis (Dunker et al., 2020). Immunoprecipitation of host AGO1 protein revealed 133 unique pathogen sRNAs, 34 of which were predicted to target at least one Arabidopsis mRNA based on stringent target prediction criteria. To establish sequence-specific targeting, the authors devised a clever assay that indirectly led to the expression of a beta-glucuronidase (GUS) reporter gene upon cleavage of a target sequence. This assay was used to test two *H. arabidopsidis* sRNAs. When the target sequence was scrambled, no GUS expression was observed, whereas a complementary target sequence led to GUS expression that extended multiple cell layers away from the *H. arabidopsidis* hyphae, suggesting that these sRNAs are secreted and mobile (Dunker et al., 2020). Furthermore, overexpression of a short tandem target site mimic, which was expected to sequester complementary sRNAs, increased the transcript levels of the target genes and rendered Arabidopsis plants more resistant to infection, showing that these sRNAs contribute to virulence inside the host cell.

A second example of an oomycete sRNA being translocated to host cells comes from work on *P. infestans* (Hu et al., 2022). The authors immunoprecipitated the *P. infestans* AGO1 protein from infected potato (*Solanum tuberosum*) leaves rather than the host AGO1 protein. Among the co-precipitated sRNAs was a miRNA-like RNA named miR8788-3p. This sRNA was predicted to target the potato gene *StABH1*, which encodes an alpha/beta hydrolase of unknown function. Evidence that miR8788-3p is translocated is indirect: *StABH1* transcript levels decreased during infection by *P. infestans*, but not when miR8788 levels were reduced in *P. infestans* using a target site mimic. Furthermore, these knockdown strains displayed reduced virulence on potato. It remains unclear, however, whether miR8788-3p is translocated as a complex with *P. infestans* AGO1 or if it also associates with the host’s AGO1.

### Spray-induced gene silencing provides mechanistic insights into HIGS

**Figure 2:**
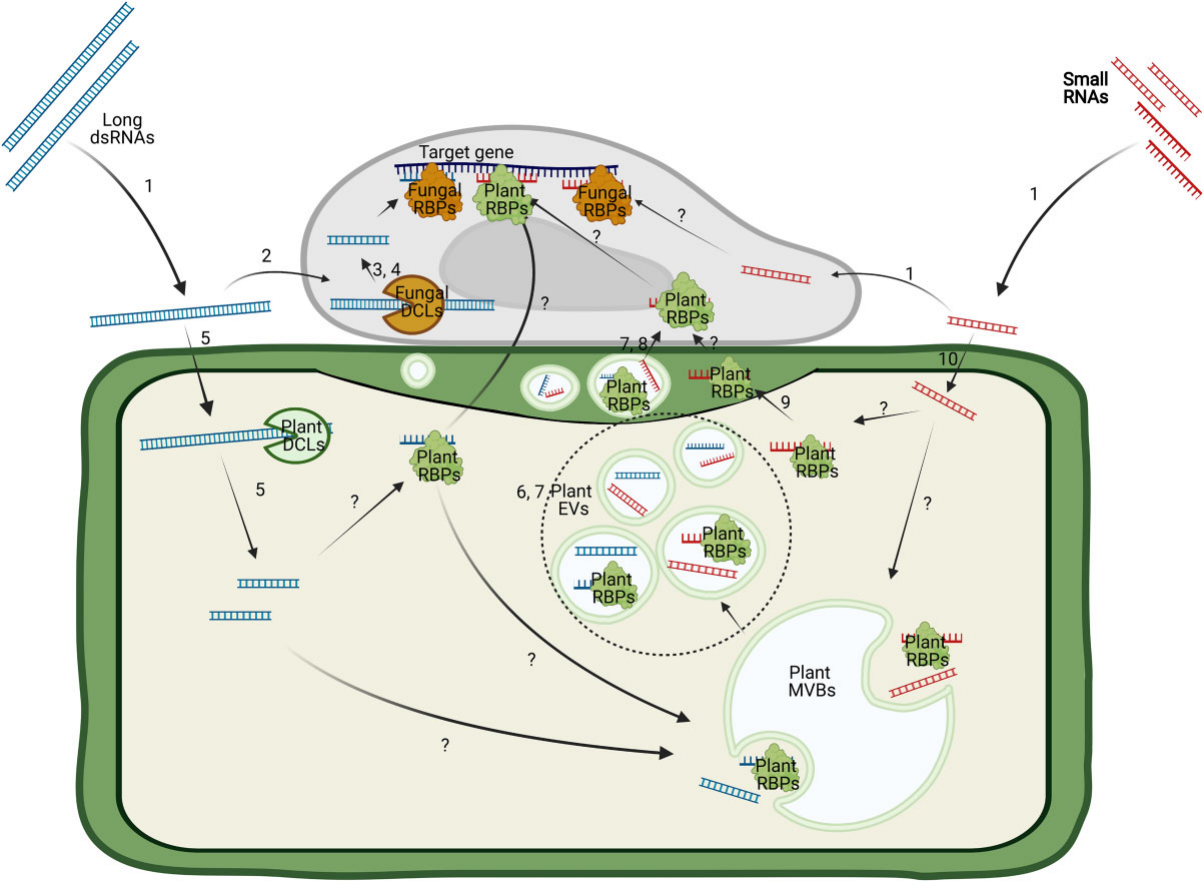
Summary of key findings related to SIGS. 1: SIGS can be induced by both long dsRNAs and sRNAs (Koch et al., 2016; Wang et al., 2016); 2: Long dsRNAs can be taken up directly by pathogens and induce gene silencing (Koch et al., 2016; Wang et al., 2016; Hu et al., 2020; Qiao et al., 2021); 3: Pathogen DCLs are essential for SIGS induced by long dsRNAs (Koch et al., 2016); 4: Pathogen DCLs process long dsRNAs into sRNAs (Koch et al., 2016); 5: Sprayed long dsRNAs can be taken up by plant cells and processed by plant DCLs (Biedenkopf et al., 2020); 6: Plant EVs associate with single-stranded sRNAs (Baldrich et al., 2019); 7: Plant EVs can deliver sRNAs into pathogen cells (Cai et al., 2018); 8: Plant RBPs bind to sRNAs and deliver them into the EVs (He et al., 2021); 9: Plant RBPs bind to sRNAs and deliver them to the apoplastic fluid (Zand Karimi et al., 2022); 10: Sprayed sRNAs can be taken up by plant cells (Dalakouras et al., 2016).

Although HIGS has shown great potential for controlling pests and diseases in crop plants, public concern about genetically modified organisms (GMOs) and restrictions on releasing GMOs into the environment have encouraged plant scientists to develop new approaches for delivering RNAs into pathogens. In a pioneering study, Koch et al. (2016) demonstrated an effective RNA spraying method, called spray-induced gene silencing (SIGS), for controlling *F.*  *graminearum* infections on barley (Figure 2). In this study, the authors sprayed a long dsRNA (CYP3-dsRNA) that targeted three fungal cytochrome P450 lanosterol C-14α-demethylase genes required for the biosynthesis of fungal ergosterol. Unexpectedly, they observed efficient spray-induced control of fungal infection in distal (nonsprayed) parts of detached leaves.

The observation of fungal gene silencing in distal tissues suggested that dsRNAs were being absorbed by the plant and that dsRNAs, or siRNAs derived from these dsRNAs, were translocating within the plant. Analysis of fluorescently labeled dsRNAs and RNA gel-blot analyses revealed that dsRNA was indeed moving intact, initially being taken up by xylem vessels in the cut surface of detached leaves but then translocating into the symplasm, including the cytoplasm of mesophyll cells (Koch et al., 2016). Importantly, silencing of target genes in the fungus inoculated on distal (nonsprayed) tissues required the fungal *DICER-LIKE 1* gene, which strongly indicates that the fungus was taking up long dsRNA from the plant and that this dsRNA was then triggering the RNAi machinery within the fungus (Koch et al., 2016). Notably, the requirement for the fungal *DCL-1* gene could be bypassed by spraying sRNAs instead of long dsRNAs (Koch et al., 2016), which indicates that *F. graminearum* can also take up sRNAs that likely then engage fungal AGO proteins.

These promising results led to numerous studies on spray application of noncoding RNAs to induce gene silencing in various organisms, including the fungal pathogens *F. graminearum* (Koch et al., 2016; Gaffar et al., 2019; Werner et al., 2020), *Phakopsora pachyrhizi* (Hu et al., 2020), *Botrytis* and *Verticillium* spp*.* (Wang et al., 2016), and insect pests such as Colorado potato beetle (San Miguel and Scott, 2016) and aphids (Biedenkopf et al., 2020; Yan et al., 2020). These studies have provided additional mechanistic insights into the movement of siRNAs and dsRNAs within leaves. For example, Biedenkopf et al. (2020) demonstrated that fluorescently labeled dsRNAs could be taken up through the stomata of barley leaves into the apoplast (extracellular space) following spray application and translocated into the phloem where they were able to move into distal, nonsprayed, portions of detached leaves, as well as into the roots of whole plants (Biedenkopf et al., 2020). Importantly, both siRNAs derived from the sprayed dsRNAs and intact dsRNAs were detected in distal phloem fluids collected from aphid stylets. This finding indicates that dsRNAs sprayed on the leaf surface can be taken up by plant cells, processed into siRNAs, and the siRNAs translocated through the phloem. Consistent with this notion, target genes in aphids were effectively silenced when aphids fed on distal portions of barley leaves, demonstrating that silencing RNAs can move systemically in barley leaves. In an independent study, Song et al. (2018) showed that dsRNA applied to cut wheat coleoptiles was efficiently taken up and processed into siRNAs. Notably, the application of dsRNAs in this manner was more efficient at silencing target genes in the fungus *F. asiaticum* than mixing dsRNAs directly into the fungal growth medium immediately prior to the inoculation of wheat coleoptiles. This observation suggests that SIGS functions via the uptake of RNAs from plant tissues rather than the direct uptake of dsRNAs from the leaf surface by the fungus. If this is indeed the case, SIGS efficacy should be enhanced by treatments that promote the uptake of dsRNAs from the leaf surface into plant cells.

One approach to promoting the uptake of exogenous RNA is to attach the RNA to nanoparticles, which are thought to protect RNAs from degradation and possibly promote endocytosis. In a recent study, however, Zhang et al. (2022) demonstrated that the delivery of siRNA bound to gold nanoparticles (AuNSs) does not require the internalization of gold nanoparticles. The infiltration of 10-nm spherical AuNSs loaded with siRNAs into the leaves of *N.*  *benthamiana* plants induced target gene silencing. Notably, the injected AuNSs were associated with cell walls or localized within the extracellular space, but not inside cells, indicating that the siRNAs were released from the nanoparticles prior to uptake. Regardless, it is clear that plant cells can take up exogenous RNA from the extracellular space, which suggests that plants employ RNA in intercellular communication.

Although SIGS appears to be a promising strategy, questions remain about its underlying mode of action, overall efficacy, and reproducibility (Dalakouras et al., 2020). For instance, Biedenkopf et al. (2020) reported efficient SIGS of the aphid (*S.*  *avenae*) *Shp* gene (encoding a structural sheath protein) when applying dsRNA to detached barley leaves. However, Liu et al. (2021) reported that the application of naked dsRNA on barley plants was inefficient in silencing different target genes (microphage migration inhibitory factor genes *MIF1, MIF2*, and *MIF3*) in the same aphid species, even though nymphs fed an artificial diet containing this dsRNA showed efficient silencing. Furthermore, the authors were unable to detect transport of fluorescently labeled dsRNA into phloem cells, contradicting the findings of Biedenkopf et al. (2020). Why these laboratories obtained conflicting results is unclear.

### HIGS appears to be part of the endogenous plant immune system

The examples of HIGS and SIGS described above involved the expression or application of artificial RNA constructs. However, there is compelling evidence that plants also translocate sRNAs from their cells to those of pathogens and pests as part of their normal immune responses. For example, Zhang et al. (2016a) showed that cotton plants upregulate two specific miRNAs when infected by the fungus *Verticillium dahliae*, miR166 and miR159. These miRNAs are taken up by the fungus, resulting in the silencing of two endogenous genes required for virulence. *Verticillium*  *dahliae* strains expressing versions of these genes in which the miRNA target sites were modified to no longer base-pair with the miRNAs exhibited enhanced virulence, demonstrating that these miRNAs contribute directly to plant immunity by targeting fungal genes. Although an exciting finding, this work raises questions as to why mutations in these target sites in the fungus have not been selected over the course of evolution given that miR166 and miR159 both function in plant development and are highly conserved across angiosperms (Achard et al., 2004; Williams et al., 2005).

A second example of “natural HIGS” comes from work in the Jin laboratory demonstrating the silencing of genes in the fungus *B. cinerea* by specific Arabidopsis siRNAs (Cai et al., 2018; He et al., 2021). The authors isolated *B. cinerea* protoplasts from infected Arabidopsis leaves and subjected the protoplasts to sRNA-seq, which revealed numerous plant siRNAs. These plant siRNAs did not appear to be contaminants, as many of the most abundant siRNAs found inside *B. cinerea* protoplasts were not among the most abundant leaf siRNAs, indicating that there was specificity in the siRNAs selected for translocation. Consistent with this hypothesis, these enriched siRNAs were found to be associated with EVs isolated from apoplastic wash fluid (AWF; Cai et al., 2018). Furthermore, disrupting siRNA biogenesis by mutating *RNA-DEPENDENT RNA POLYMERASE* (*RDR6*) increased susceptibility to *B. cinerea*, suggesting that siRNAs play a direct role in immunity. In support of this conclusion, predicted target genes of these siRNAs in *B. cinerea* were downregulated during infection, but not in *B. cinerea* collected from infected *rdr6* mutant plants (Cai et al., 2018).

As a third example of HIGS involving endogenous sRNAs, Hou et al. (2019) showed that infecting Arabidopsis with the oomycete pathogen *Phytophthora capsici* induced the production of a diverse pool of siRNAs, at least some of which were secreted to the extracellular space*.* Importantly, disrupting siRNA biogenesis rendered Arabidopsis hypersusceptible to infection by *P. capsici*, and transgenic expression of specific siRNAs in this pathogen inhibited its virulence, suggesting that secreted siRNAs contribute directly to plant immunity. Consistent with this notion, a potential target gene in *P. capsici* of one specific siRNA was downregulated during infection in wild-type plants, but not in plants compromised in siRNA production (Hou et al., 2019).

Likely related to HIGS is the recent observation of sRNA transfer from parasitic plants into their hosts (Shahid et al., 2018), although strictly speaking this would be considered pathogen-induced gene silencing. In this study, several Arabidopsis mRNAs were shown to be targeted by microRNAs produced by the parasitic plant *Cucusta campestris.* These microRNAs moved into host cells during infection, with the target mRNAs exhibiting reduced accumulation. Furthermore, knockout of these genes rendered Arabidopsis significantly more susceptible to *C. campestris* than the control, indicating that *C. campestris* miRNAs play a central role in parasitism by silencing target genes in the host.

### Extracellular RNAs

**Figure 3:**
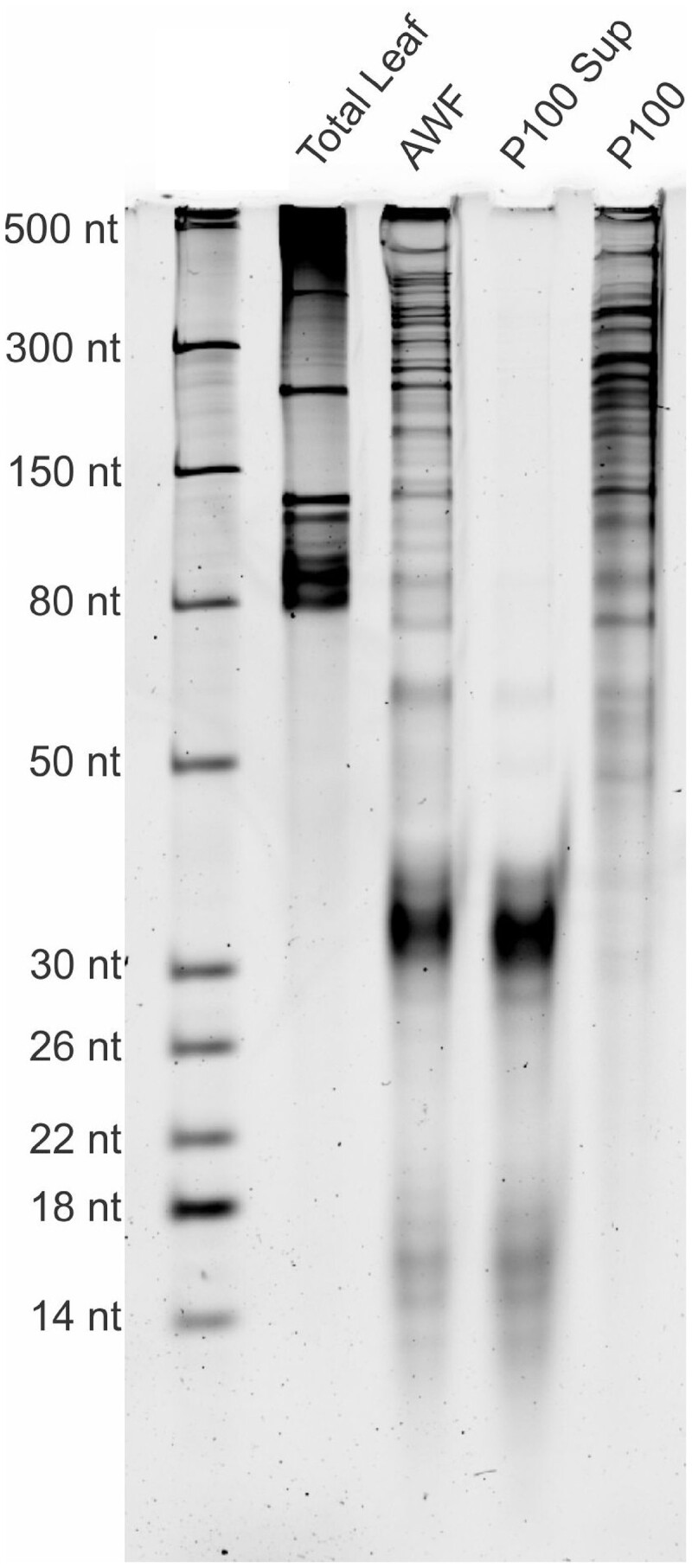
AWF contains diverse RNA species. Image taken from Zand Karimi (2022). Shown is a polyacrylamide denaturing RNA gel stained with SYBR Gold fluorescent nucleic acid stain. RNA isolated from AWF displays a size distribution completely different from total leaf lysate. RNAs longer than ∼40 nt are nearly all pelleted by centrifugation at 100,000*g* for 1 h (P100). Most smaller RNAs remain in the supernatant (P100 Sup).

The above studies indicated that plants secrete sRNAs into the apoplast but did not provide comprehensive analysis of apoplastic RNA. To address this gap in knowledge, several groups have pursued in-depth analyses of apoplastic RNAs (Baldrich et al., 2019; Tosar and Cayota, 2020; Zand Karimi et al., 2022). Analyses of apoplastic RNA isolated from Arabidopsis leaves have revealed abundant RNA species ranging in size from ∼10 nt to >500 nt (Figure 3; Zand Karimi et al., 2022). The great majority of apoplastic RNAs longer than 40 nt could be pelleted by centrifuging the AWF at 100,000*g*, indicating that long RNAs are associated with particles of some kind. Conversely, the majority of smaller RNAs could not be pelleted, suggesting that they are not associated with particles, including plant EVs, which should all pellet at 100,000*g* (Rutter and Innes, 2017). Based on protease and RNase protection assays on P100 pellets (materials pelleted by centrifugation at 100,000*g* for 1 h), the RNAs in these pellets were found to be located outside EVs (Zand Karimi et al., 2022), suggesting that plant EVs do not carry significant amounts of RNA. This conclusion is consistent with quantitative analyses of miRNAs in human EVs, which found that even the most abundant miRNAs are present at concentrations of far less than one miRNA molecule per vesicle, with the majority of extracellular miRNAs located outside of EVs (Arroyo et al., 2011; Chevillet et al., 2014). Based on these findings and those of Zand Karimi et al. (2022), we believe that it is unlikely that EVs play a direct role in the translocation of endogenous RNAs from plant cells to pathogens.

This conclusion runs counter to conclusions reached by Cai et al. (2018) and He et al. (2021), who reported that siRNAs taken up by *B. cinerea* were carried inside EVs. The authors showed that siRNAs purified from the apoplast were protected against degradation by micrococcal nuclease but not when the pellet was pretreated with detergent that should disrupt EV membranes. However, work in our laboratory (Baldrich et al., 2019; Zand Karimi et al., 2022) has shown that most apoplastic siRNAs do not co-pellet with EVs (Figure 3), and those that do can be degraded by RNase A if the pellet is pretreated with protease, even though protease treatment does not disrupt EVs. Based on these observations, we concluded that some siRNAs are packaged inside protein complexes that co-purify with EVs, but these are not located inside EVs. It is possible that the detergent treatments described in Cai et al. (2018) disrupted protein–RNA complexes as well as EVs, thus rendering the siRNAs sensitive to nuclease digestion. It should be noted, however, that these methods represent indirect assays for the localization of siRNAs. Final resolution of this debate may require in situ analyses using super resolution or electron microscopy.

Analysis of apoplastic RNAs by RNA-seq have shown that the majority of these RNAs are derived from ribosomal RNA and intergenic regions (Zand Karimi et al., 2022). Notably, apoplastic RNAs also contain a large number of circular RNAs (Zand Karimi et al., 2022), which were recently shown to contribute to immune responses in rice against the fungal pathogen *M. oryzae* (Fan et al., 2020). We thus speculate that extracellular long RNAs, especially circular RNAs, play important roles in plant–microbe interactions, representing an important issue for further study.

Zand Karimi et al. (2022) revealed that the majority of apoplastic RNA is protected against degradation via association with extracellular proteins. Consistent with this idea, multiple RNA-binding proteins have been identified in AWFs (He et al., 2021), including AGO2 and GLYCINE-RICH RNA-BINDING PROTEIN 7 (GRP7; Zand Karimi et al., 2022). Notably, Arabidopsis *grp7* and *ago2* single mutants displayed significant changes in apoplastic RNA contents relative to wild-type plants, suggesting that these two proteins contribute directly or indirectly to the secretion and/or stabilization of extracellular RNAs (Zand Karimi et al., 2022). We thus hypothesize that extracellular RNA–protein complexes are the primary mediators of HIGS.

If our hypothesis is correct, several interesting questions arise: How are specific RNA–protein complexes selected for secretion into the apoplast, and how are these complexes taken up by pathogens? Recently, He et al. (2021) identified several RNA-binding proteins in the apoplastic fluids of Arabidopsis, including AGO1, RNA helicases (RH11 and RH37), and annexins (ANN1 and ANN2). They also reported that pathogen infection increases the secretion of these RNA binding proteins (RBPs) into the apoplast. Using protease protection assays, the authors showed that several of these proteins are protected against protease digestion in the absence of detergent, suggesting that they are located inside EVs. How these proteins are encapsulated inside EVs, and whether EVs release these proteins inside pathogens, are not yet known. These data suggest that these specific RNA-binding proteins contribute to HIGS, but this hypothesis remains to be tested.

### RNA uptake by pathogens and pests

How RNAs move from plant cells into the cells of a pathogen or pest is unclear, but this process likely depends on the pathogen’s lifestyle (Koch and Wassenegger, 2021). As described above, insects and nematodes take up plant RNAs from plant cells using their feeding structures, delivering RNA into their digestive systems. Somehow this RNA can survive in the digestive system and be absorbed by intestinal epithelial cells, and from there the RNAi signal spreads into distal tissues via poorly understood mechanisms (Tian et al., 2016; Biedenkopf et al., 2020). Although we have little understanding of RNA uptake by fungi, the observation that some fungal pathogens can take up naked sRNA and long dsRNAs from their environment suggest that RNAs might be taken up via an endocytic process (Wang et al., 2016; Qiao et al., 2021). How the endocytosed RNA is then released intact from endosomes to engage the fungal RNAi machinery represents a major question about the HIGS process that remains to be answered.

### Phloem RNA

In addition to the apoplast, another likely location for RNA transfer from plants to pathogens and pests is the phloem. Zhang et al. (2009b) assessed the RNA content of phloem in pumpkin (*Cucurbita maxima*) and identified several species of RNA, including siRNAs, microRNAs, spliceosomal RNAs, and tRNA fragments. The latter were shown to interfere with translation. Similar results were reported by Lalande et al. (2020), who found that 30–35-nt and 19–25-nt tRNA fragments from Arabidopsis inhibited the in vitro translation of reporter genes. These observations raise the interesting possibility that tRNA fragments also inhibit translation in pathogens and pests when taken up from the phloem or apoplast. It has been suggested that phloem-mobile mRNAs might contain sequence elements in their UTRs that adopt tRNA-like structures that direct RNA into the phloem (Kehr and Buhtz, 2008). Indeed, tRNA-like structures have been detected in viral RNAs that translocate through phloem (Lezzhov et al., 2019).

Long-distance RNA translocation through the phloem was confirmed by assessing sRNA/mRNA movement across graft junctions (Ruiz-Medrano et al., 1999; Zhang et al., 2009a; Azumi Kanehira et al., 2010; Notaguchi et al., 2012; Thieme et al., 2015; Zhao et al., 2020). Similarly, mRNA movement through the phloem has been reported in the host–parasite junctions of parasitic plants (David-Schwartz et al., 2008). Interestingly, it appears that RNAs in the phloem are safe from degradation because there is no detectable RNase activity in phloem sap (Sasaki et al., 1998; Doering-Saad et al., 2002; Kehr and Buhtz, 2008).

Although diverse RNA species have been detected in both the phloem (David-Schwartz et al., 2008) and apoplast (Baldrich et al., 2019; Zand Karimi et al., 2022), the mechanism of RNA translocation between the symplast, apoplast, and phloem is not yet clear. Studies on the movement of sRNAs between the maternal tissues, endosperm, and embryos of Arabidopsis showed that sRNAs can move from endosperm cells to embryo cells, even though there are no direct connections between these cells (Martienssen, 2010). This observation indicates that sRNAs must transit through the apoplast in developing seeds (Martienssen, 2010; Melnyk et al., 2011).

Additional evidence for the apoplastic transport of RNA comes from a study in which hpRNAs and siRNAs were directly injected into the trunks of grapevine (*Vitis vinifera*) and introduced through the base of cut petioles into *N. benthamiana* (Dalakouras et al., 2018). These experiments showed that both classes of RNA can spread systemically in plants through the xylem without significant degradation over the course of 10 days. Notably, petiole absorption of siRNA labeled with the fluorescent dye CY3 produced fluorescent signal in the guard cells of systemic leaves, but not in mesophyll or other epidermal cells. Since guard cells lack plasmodesmata (Wille and Lucas, 1984), they likely took up RNA directly from the apoplast. Why only guard cells were able to take up RNA from the apoplast is unclear. Consistent with the lack of uptake by mesophyll cells, no silencing of a target GFP transgene was observed.

### Viruses as tools for studying RNA transport

Plant RNA viruses can spread systemically in the host, which represents a form of intercellular RNA transport. RNA movement appears to occur through both the symplast and apoplast (Omid et al., 2008; Wan and Laliberté, 2015; Movahed et al., 2019). For symplastic movement, a key step is passage through plasmodesmata, which are often modified by viral proteins (Sambade et al., 2008; Epel, 2009). Apoplastic movement is less well-characterized, but Movahed et al. (2019) reported the release of turnip mosaic virus (TuMV) RNA into the apoplast via fusion of multi-vesicular bodies with the plasma membrane. Whether viral RNAs can use EVs to move between cells is not yet clear, but viral RNA fragments can be detected in apoplastic fluid (Movahed et al., 2019; Hu et al., 2021). Although Movahed et al. reported the presence of TuMV viral particles associated with EVs, Hu et al. (2021) reported that PVX particles found in apoplastic fluid were not associated with EVs. The functional significance of apoplastic viral RNA thus remains enigmatic.

### Apoplastic RNases

The apoplast contains several species of extracellular ribonucleases (ex-ribonucleases), which have been shown to target viral RNAs (Sangaev et al., 2007; Sugawara et al., 2016; Potrokhov et al., 2021). Some of these ex-ribonucleases are induced in response to wounding and viral infection (Kurata et al., 2002; LeBrasseur et al., 2002). The expression of a wound-induced ex-ribonuclease from *Zinnia elegans* enhanced resistance to potato virus Y in tobacco plants, confirming a role for this ex-ribonuclease in virus resistance. Whether these ex-ribonucleases also affect apoplastic RNAs of plant origin is not clear.

Some fungal pathogens also secrete ribonuclease-like effectors, presumably to target plant-derived RNAs (Pliego et al., 2013; Pennington et al., 2019). However, these effectors appear to lack catalytic activity and thus may function as RBPs rather than RNA degrading enzymes. How dsRNAs/sRNAs are protected against plant and pathogen ribonucleases in the apoplast is unclear, but the presence of RNA-binding proteins in the apoplast suggest that these RNAs may be protected by these proteins (He et al., 2021; Zand Karimi et al., 2022).

### Potential roles of RNA-binding proteins in RNA movement

Proteomic analyses of phloem sap, xylem sap, and AWFs have shown that these three fluids share overlapping sets of proteins, including multiple RNA-binding proteins (Pallas and Gómez, 2013; Rodríguez-Celma et al., 2016; Rutter and Innes, 2017; Godson and van der Hoorn, 2021). For example, Rutter and Innes (2017) reported that plant EVs contain heat shock proteins, annexins, and a member of the small RBP family (GRP7). Remarkably, some of these proteins appear to be involved in the transport of sRNAs or viral RNAs between cells. Yan et al. (2020) showed that Arabidopsis GRP7 mediates the movement of sRNAs between cells through the plasmodesmata. They also noted that GRP7 binds to single-stranded viral RNAs and transfers them between cells by interacting with plasmodesmatal receptors (Yan et al., 2020). GRP7 belongs to the same family of RNA-binding proteins as HNRNPA2B1 in mammalian cells, which plays a role in loading sRNAs into EVs and in viral RNA movement in mammalian cells (Villarroya-Beltri et al., 2013; Zhou et al., 2020).

Similarly, several RNA-binding proteins in phloem sap have been reported to play critical roles in long-distance RNA translocation (Xoconostle-Cázares et al., 1999; Yoo et al., 2004; Yan et al., 2020). Yoo et al. (2004) identified *Cm*PSRP1 as an RNA-binding protein that binds to siRNAs in the phloem of pumpkin and facilitates intercellular movement. Interestingly, a recent study on siRNA movement in Arabidopsis revealed that siRNAs move long distance and cell-to-cell as double-stranded rather than single-stranded siRNAs and are not bound to AGO proteins (Devers et al., 2020), suggesting that there are specific dsRNA-binding proteins that mediate both local and long-distance siRNA movement.

The above examples mostly focus on the intra-organismal movement of RNAs. However, RNA-binding proteins likely play important roles in inter-organismal movement as well. An interesting example of this has been described in honeybee colonies, where diverse protein-coding and noncoding RNAs are secreted into royal jelly by worker bees and fed to developing larvae in the hive (Maori et al., 2019). These RNAs are stabilized by an RNA-binding protein named Major Royal Jelly Protein 3 (MRJP-3), which makes up 10%–15% of total proteins in royal jelly. MRJP-3 binds to both single-stranded RNA and dsRNA with a minimum length of 18 nt. This binding occurs in a multivalent fashion, which leads to the formation of large protein-RNA aggregates that are resistant to degradation by RNase A. Notably, MRJP-3 was shown to enhance the uptake of dsRNAs by the nematode *C.*  *elegans*, suggesting that one of its functions is to enhance the transmission of RNAs from worker bees to larvae (Maori et al., 2019). We speculate that plants may secrete RNA-binding proteins such as GRP7 into the apoplast to enhance the uptake of plant RNAs by microbes.

### Future directions for foundational HIGS and SIGS research

Additional investigations are required to understand the mechanisms of RNA uptake by both plants and pathogens, as these insights should lead to improvements in the efficacy of both HIGS and SIGS. Basic questions remain to be answered, such as how specific RNAs and RNA-binding proteins are selected for export, and how they are exported. The roles of pathogen RNAi machineries in HIGS and SIGS also need to be determined. Does SIGS require specific AGO proteins from plants or pathogens? What is the mechanism of RNA uptake and translocation by pathogens? Once RNA is taken up by a pathogen, how is it released into the cytoplasm to engage with pathogen RNAs? Addressing this long list of questions will require a robust HIGS system in which candidate genes can be disrupted in both the host and pathogen, allowing individual genes and gene families to be assessed for their contributions to HIGS and SIGS.

## Notes

H.Z.K. and R.W.I. outlined, wrote, and edited this review.

The author responsible for distribution of materials integral to the findings presented in this article in accordance with the policy described in the Instructions for Authors (https://academic.oup.com/plcell) is: Roger W. Innes (rinnes@indiana.edu).

## References

[koac165-B1] Abdellatef E , Will T, Koch A, Imani J, Vilcinskas A, Kogel KH (2015) Silencing the expression of the salivary sheath protein causes transgenerational feeding suppression in the aphid *Sitobion avenae*. Plant Biotechnol J 13: 849–857 25586210 10.1111/pbi.12322

[koac165-B2] Achard P , Herr A, Baulcombe DC, Harberd NP (2004) Modulation of floral development by a gibberellin-regulated microRNA. Development 131: 3357–3365 15226253 10.1242/dev.01206

[koac165-B3] Ahmed F , Senthil-Kumar M, Dai X, Ramu VS, Lee S, Mysore KS, Zhao PX (2020) pssRNAit: a web server for designing effective and specific plant siRNAs with genome-wide off-target assessment. Plant Physiol 184: 65–81 32651189 10.1104/pp.20.00293 PMC7479913

[koac165-B4] Alakonya A , Kumar R, Koenig D, Kimura S, Townsley B, Runo S, Garces HM, Kang J, Yanez A, David-Schwartz R, et al (2012) Interspecific RNA interference of SHOOT MERISTEMLESS-like disrupts *Cuscuta pentagona* plant parasitism. Plant Cell 24: 3153–3166 22822208 10.1105/tpc.112.099994 PMC3426138

[koac165-B5] Antao VP , Tinoco I Jr (1992) Thermodynamic parameters for loop formation in RNA and DNA hairpin tetraloops. Nucleic Acids Res 20: 819–824 1371866 10.1093/nar/20.4.819 PMC312023

[koac165-B6] Arroyo JD , Chevillet JR, Kroh EM, Ruf IK, Pritchard CC, Gibson DF, Mitchell PS, Bennett CF, Pogosova-Agadjanyan EL, Stirewalt DL, et al (2011) Argonaute2 complexes carry a population of circulating microRNAs independent of vesicles in human plasma. Proc Natl Acad Sci USA 108: 5003–5008 21383194 10.1073/pnas.1019055108 PMC3064324

[koac165-B7] Azumi Kanehira KY , Tomomi Iwaya R, Tsuwamoto AK, Mikio N, Harada T (2010) Apple phloem cells contain some mRNAs transported over long distances. Tree Genet Genom 6: 635–642

[koac165-B8] Bakhetia M , Charlton W, Atkinson HJ, McPherson MJ (2005) RNA interference of dual oxidase in the plant nematode *Meloidogyne incognita*. Mol Plant Microbe Interact 18: 1099–1106 16255249 10.1094/MPMI-18-1099

[koac165-B9] Baldrich P , Rutter BD, Karimi HZ, Podicheti R, Meyers BC, Innes RW (2019) Plant extracellular vesicles contain diverse small RNA species and are enriched in 10- to 17-nucleotide “Tiny” RNAs. Plant Cell 31: 315–324 30705133 10.1105/tpc.18.00872 PMC6447009

[koac165-B10] Bally J , Fishilevich E, Doran RL, Lee K, de Campos SB, German MA, Narva KE, Waterhouse PM (2020) Plin-amiR, a pre-microRNA-based technology for controlling herbivorous insect pests. Plant Biotechnol J 18: 1925–1932 32012433 10.1111/pbi.13352 PMC7415779

[koac165-B11] Bally J , McIntyre GJ, Doran RL, Lee K, Perez A, Jung H, Naim F, Larrinua IM, Narva KE, Waterhouse PM (2016) In-plant protection against *Helicoverpa armigera* by production of long hpRNA in chloroplasts. Front Plant Sci 7: 1453 27746796 10.3389/fpls.2016.01453 PMC5040858

[koac165-B12] Baumberger N , Baulcombe DC (2005) Arabidopsis ARGONAUTE1 is an RNA Slicer that selectively recruits microRNAs and short interfering RNAs. Proc Natl Acad Sci USA 102: 11928–11933 16081530 10.1073/pnas.0505461102 PMC1182554

[koac165-B13] Bernstein E , Caudy AA, Hammond SM, Hannon GJ (2001) Role for a bidentate ribonuclease in the initiation step of RNA interference. Nature 409: 363–366 11201747 10.1038/35053110

[koac165-B14] Biedenkopf D , Will T, Knauer T, Jelonek L, Furch ACU, Busche T, Koch A (2020) Systemic spreading of exogenous applied RNA biopesticides in the crop plant *Hordeum vulgare*. ExRNA 2: 12

[koac165-B35291486] **Boutla A, Kalantidis K, Tavernarakis N, Tsagris M, Tabler M** (2002) Induction of RNA interference in Caenorhabditis elegans by RNAs derived from plants exhibiting post-transcriptional gene silencing. Nucleic Acids Res **30**: 1688–1694 10.1093/nar/30.7.1688 PMC101830 11917031

[koac165-B15] Brosseau C , Moffett P (2015) Functional and genetic analysis identify a role for Arabidopsis ARGONAUTE5 in antiviral RNA silencing. Plant Cell 27: 1742–1754 26023161 10.1105/tpc.15.00264 PMC4498209

[koac165-B16] Cai Q , Qiao L, Wang M, He B, Lin FM, Palmquist J, Huang SD, Jin H (2018) Plants send small RNAs in extracellular vesicles to fungal pathogen to silence virulence genes. Science 360: 1126–1129 29773668 10.1126/science.aar4142 PMC6442475

[koac165-B17] Campo S , Gilbert KB, Carrington JC (2016) Small RNA-based antiviral defense in the phytopathogenic fungus *Colletotrichum higginsianum*. PLoS Pathog 12: e1005640 27253323 10.1371/journal.ppat.1005640 PMC4890784

[koac165-B18] Cappelle K , de Oliveira CF, Van Eynde B, Christiaens O, Smagghe G (2016) The involvement of clathrin-mediated endocytosis and two Sid-1-like transmembrane proteins in double-stranded RNA uptake in the Colorado potato beetle midgut. Insect Mol Biol 25: 315–323 26959524 10.1111/imb.12222

[koac165-B19] Carbonell A (2017) Plant ARGONAUTEs: features, functions, and unknowns. Methods Mol Biol 1640: 1–21 28608331 10.1007/978-1-4939-7165-7_1

[koac165-B20] Chevillet JR , Kang Q, Ruf IK, Briggs HA, Vojtech LN, Hughes SM, Cheng HH, Arroyo JD, Meredith EK, Gallichotte EN, et al (2014) Quantitative and stoichiometric analysis of the microRNA content of exosomes. Proc Natl Acad Sci USA 111: 14888–14893 25267620 10.1073/pnas.1408301111 PMC4205618

[koac165-B21] Cogoni C , Macino G (1997) Isolation of quelling-defective (qde) mutants impaired in posttranscriptional transgene-induced gene silencing in *Neurospora crassa*. Proc Natl Acad Sci USA 94: 10233–10238 9294193 10.1073/pnas.94.19.10233 PMC23345

[koac165-B22] Cogoni C , Macino G (1999) Gene silencing in *Neurospora crassa* requires a protein homologous to RNA-dependent RNA polymerase. Nature 399: 166–169 10335848 10.1038/20215

[koac165-B23] Dalakouras A , Wassenegger M, Dadami E, Ganopoulos I, Pappas ML, Papadopoulou K (2020) Genetically modified organism-free RNA interference: exogenous application of RNA molecules in plants. Plant Physiol 182: 38–50 31285292 10.1104/pp.19.00570 PMC6945881

[koac165-B24] Dalakouras A , Jarausch W, Buchholz G, Bassler A, Braun M, Manthey T, Krczal G, Wassenegger M (2018) Delivery of hairpin RNAs and small RNAs into woody and herbaceous plants by trunk injection and petiole absorption. Front Plant Sci 9: 1253 30210521 10.3389/fpls.2018.01253 PMC6120046

[koac165-B25] Dalakouras A , Wassenegger M, McMillan JN, Cardoza V, Maegele I, Dadami E, Runne M, Krczal G, Wassenegger M (2016) Induction of silencing in plants by high-pressure spraying of in vitro-synthesized small RNAs. Front Plant Sci 7: 1327 27625678 10.3389/fpls.2016.01327 PMC5003833

[koac165-B26] David-Schwartz R , Runo S, Townsley B, Machuka J, Sinha N (2008) Long-distance transport of mRNA via parenchyma cells and phloem across the host-parasite junction in Cuscuta. New Phytol 179: 1133–1141 18631294 10.1111/j.1469-8137.2008.02540.x

[koac165-B27] Devers EA , Brosnan CA, Sarazin A, Albertini D, Amsler AC, Brioudes F, Jullien PE, Lim P, Schott G, Voinnet O (2020) Movement and differential consumption of short interfering RNA duplexes underlie mobile RNA interference. Nat Plants 6: 789–799 32632272 10.1038/s41477-020-0687-2

[koac165-B28] Doering-Saad C , Newbury HJ, Bale JS, Pritchard J (2002) Use of aphid stylectomy and RT-PCR for the detection of transporter mRNAs in sieve elements. J Exp Bot 53: 631–637 11886882 10.1093/jexbot/53.369.631

[koac165-B29] Dong Y , Friedrich M (2005) Nymphal RNAi: systemic RNAi mediated gene knockdown in juvenile grasshopper. BMC Biotechnol 5: 25 16202143 10.1186/1472-6750-5-25 PMC1266053

[koac165-B30] Dunker F , Trutzenberg A, Rothenpieler JS, Kuhn S, Prols R, Schreiber T, Tissier A, Kemen A, Kemen E, Huckelhoven R, et al (2020) Oomycete small RNAs bind to the plant RNA-induced silencing complex for virulence. eLife 9: e56096 32441255 10.7554/eLife.56096 PMC7297541

[koac165-B31] Eamens A , Wang MB, Smith NA, Waterhouse PM (2008) RNA silencing in plants: yesterday, today, and tomorrow. Plant Physiol 147: 456–468 18524877 10.1104/pp.108.117275 PMC2409047

[koac165-B32] Elbashir SM , Martinez J, Patkaniowska A, Lendeckel W, Tuschl T (2001) Functional anatomy of siRNAs for mediating efficient RNAi in *Drosophila melanogaster* embryo lysate. EMBO J 20: 6877–6888 11726523 10.1093/emboj/20.23.6877 PMC125328

[koac165-B33] English JJ , Mueller E, Baulcombe DC (1996) Suppression of virus accumulation in transgenic plants exhibiting silencing of nuclear genes. Plant Cell 8: 179–188 12239381 10.1105/tpc.8.2.179 PMC161090

[koac165-B34] Epel BL (2009) Plant viruses spread by diffusion on ER-associated movement-protein-rafts through plasmodesmata gated by viral induced host beta-1,3-glucanases. Semin Cell Dev Biol 20: 1074–1081 19501662 10.1016/j.semcdb.2009.05.010

[koac165-B35] Fagard M , Boutet S, Morel JB, Bellini C, Vaucheret H (2000) AGO1, QDE-2, and RDE-1 are related proteins required for post-transcriptional gene silencing in plants, quelling in fungi, and RNA interference in animals. Proc Natl Acad Sci USA 97: 11650–11654 11016954 10.1073/pnas.200217597 PMC17255

[koac165-B36] Fan J , Quan W, Li GB, Hu XH, Wang Q, Wang H, Li XP, Luo X, Feng Q, Hu ZJ, et al (2020) circRNAs are involved in the rice-*Magnaporthe oryzae* interaction. Plant Physiol 182: 272–286 31628150 10.1104/pp.19.00716 PMC6945833

[koac165-B37] Feinberg EH , Hunter CP (2003) Transport of dsRNA into cells by the transmembrane protein SID-1. Science 301: 1545–1547 12970568 10.1126/science.1087117

[koac165-B38] Fusaro AF , Matthew L, Smith NA, Curtin SJ, Dedic-Hagan J, Ellacott GA, Watson JM, Wang MB, Brosnan C, Carroll BJ, et al (2006) RNA interference-inducing hairpin RNAs in plants act through the viral defence pathway. EMBO Rep 7: 1168–1175 17039251 10.1038/sj.embor.7400837 PMC1679793

[koac165-B39] Gaffar FY , Imani J, Karlovsky P, Koch A, Kogel KH (2019) Different components of the RNA interference machinery are required for conidiation, ascosporogenesis, virulence, deoxynivalenol production, and fungal inhibition by exogenous double-stranded RNA in the head blight pathogen *Fusarium graminearum*. Front Microbiol 10: 1662 31616385 10.3389/fmicb.2019.01662 PMC6764512

[koac165-B40] Gasciolli V , Mallory AC, Bartel DP, Vaucheret H (2005) Partially redundant functions of Arabidopsis DICER-like enzymes and a role for DCL4 in producing trans-acting siRNAs. Curr Biol 15: 1494–1500 16040244 10.1016/j.cub.2005.07.024

[koac165-B41] Gasparis S , Kała M, Przyborowski M, Orczyk W, Nadolska-Orczyk A (2016) Artificial microRNA-based specific gene silencing of grain hardness genes in polyploid cereals appeared to be not stable over transgenic plant generations. Front Plant Sci 7: 2017 28119710 10.3389/fpls.2016.02017 PMC5220083

[koac165-B42] Ghag SB , Shekhawat UK, Ganapathi TR (2014) Host-induced post-transcriptional hairpin RNA-mediated gene silencing of vital fungal genes confers efficient resistance against Fusarium wilt in banana. Plant Biotechnol J 12: 541–553 24476152 10.1111/pbi.12158

[koac165-B43] Godson A , van der Hoorn RAL (2021) The front line of defence: a meta-analysis of apoplastic proteases in plant immunity. J Exp Bot 72: 3381–3394 33462613 10.1093/jxb/eraa602 PMC8042752

[koac165-B44] Govindarajulu M , Epstein L, Wroblewski T, Michelmore RW (2015) Host-induced gene silencing inhibits the biotrophic pathogen causing downy mildew of lettuce. Plant Biotechnol J 13: 875–883 25487781 10.1111/pbi.12307

[koac165-B45] Groebe DR , Uhlenbeck OC (1988) Characterization of RNA hairpin loop stability. Nucleic Acids Res 16: 11725–11735 3211748 10.1093/nar/16.24.11725 PMC339106

[koac165-B46] Guo H , Song X, Wang G, Yang K, Wang Y, Niu L, Chen X, Fang R (2014) Plant-generated artificial small RNAs mediated aphid resistance. PLoS One 9: e97410 24819752 10.1371/journal.pone.0097410 PMC4018293

[koac165-B47] Guo Q , Liu Q, Smith NA, Liang G, Wang MB (2016) RNA silencing in plants: mechanisms, technologies and applications in horticultural crops. Curr Genomics 17: 476–489 28217004 10.2174/1389202917666160520103117 PMC5108043

[koac165-B48] Guo XY , Li Y, Fan J, Xiong H, Xu FX, Shi J, Shi Y, Zhao JQ, Wang YF, Cao XL, et al (2019) Host-induced gene silencing of *MoA*P1 confers broad-spectrum resistance to *Magnaporthe oryzae*. Front Plant Sci 10: 433 31024598 10.3389/fpls.2019.00433 PMC6465682

[koac165-B49] Hammond SM , Bernstein E, Beach D, Hannon GJ (2000) An RNA-directed nuclease mediates post-transcriptional gene silencing in Drosophila cells. Nature 404: 293–296 10749213 10.1038/35005107

[koac165-B50] Havecker ER , Wallbridge LM, Hardcastle TJ, Bush MS, Kelly KA, Dunn RM, Schwach F, Doonan JH, Baulcombe DC (2010) The Arabidopsis RNA-directed DNA methylation argonautes functionally diverge based on their expression and interaction with target loci. Plant Cell 22: 321–334 20173091 10.1105/tpc.109.072199 PMC2845420

[koac165-B51] He B , Cai Q, Qiao L, Huang CY, Wang S, Miao W, Ha T, Wang Y, Jin H (2021) RNA-binding proteins contribute to small RNA loading in plant extracellular vesicles. Nat Plants 7: 342–352 33633358 10.1038/s41477-021-00863-8 PMC7979528

[koac165-B52] Honeybee Genome Sequencing C (2006) Insights into social insects from the genome of the honeybee *Apis mellifera*. Nature 443: 931–949 17073008 10.1038/nature05260 PMC2048586

[koac165-B53] Hou Y , Zhai Y, Feng L, Karimi HZ, Rutter BD, Zeng L, Choi DS, Zhang B, Gu W, Chen X, et al (2019) A Phytophthora effector suppresses trans-kingdom RNAi to promote disease susceptibility. Cell Host Microbe 25: 153–165.e155 30595554 10.1016/j.chom.2018.11.007 PMC9208300

[koac165-B54] Hu D , Chen ZY, Zhang C, Ganiger M (2020) Reduction of *Phakopsora pachyrhizi* infection on soybean through host- and spray-induced gene silencing. Mol Plant Pathol 21: 794–807 32196911 10.1111/mpp.12931 PMC7214474

[koac165-B55] Hu S , Yin Y, Chen B, Lin Q, Tian Y, Song X, Peng J, Zheng H, Rao S, Wu G, et al (2021) Identification of viral particles in the apoplast of *Nicotiana benthamiana* leaves infected by potato virus X. Mol Plant Pathol 22: 456–464 33629491 10.1111/mpp.13039 PMC7938632

[koac165-B56] Hu X , Persson Hoden K, Liao Z, Asman A, Dixelius C (2022) *Phytophthora infestans* Ago1-associated miRNA promotes potato late blight disease. New Phytol 233: 443–457 34605025 10.1111/nph.17758

[koac165-B57] Huang G , Allen R, Davis EL, Baum TJ, Hussey RS (2006) Engineering broad root-knot resistance in transgenic plants by RNAi silencing of a conserved and essential root-knot nematode parasitism gene. Proc Natl Acad Sci USA 103: 14302–14306 16985000 10.1073/pnas.0604698103 PMC1570184

[koac165-B58] Jahan SN , Asman AK, Corcoran P, Fogelqvist J, Vetukuri RR, Dixelius C (2015) Plant-mediated gene silencing restricts growth of the potato late blight pathogen *Phytophthora infestans*. J Exp Bot 66: 2785–2794 25788734 10.1093/jxb/erv094 PMC4986879

[koac165-B59] Jullien PE , Grob S, Marchais A, Pumplin N, Chevalier C, Bonnet DMV, Otto C, Schott G, Voinnet O (2020) Functional characterization of Arabidopsis ARGONAUTE 3 in reproductive tissues. Plant J 103: 1796–1809 32506562 10.1111/tpj.14868

[koac165-B60] Kadotani N , Nakayashiki H, Tosa Y, Mayama S (2003) RNA silencing in the phytopathogenic fungus *Magnaporthe oryzae*. Mol Plant Microbe Interact 16: 769–776 12971600 10.1094/MPMI.2003.16.9.769

[koac165-B61] Kadotani N , Nakayashiki H, Tosa Y, Mayama S (2004) One of the two Dicer-like proteins in the filamentous fungi *Magnaporthe oryzae* genome is responsible for hairpin RNA-triggered RNA silencing and related small interfering RNA accumulation. J Biol Chem 279: 44467–44474 15304480 10.1074/jbc.M408259200

[koac165-B62] Kehr J , Buhtz A (2008) Long distance transport and movement of RNA through the phloem. J Exp Bot 59: 85–92 17905731 10.1093/jxb/erm176

[koac165-B63] Koch A , Wassenegger M (2021) Host-induced gene silencing - mechanisms and applications. New Phytol 231: 54–59 33774815 10.1111/nph.17364

[koac165-B64] Koch A , Kumar N, Weber L, Keller H, Imani J, Kogel KH (2013) Host-induced gene silencing of cytochrome P450 lanosterol C14alpha-demethylase-encoding genes confers strong resistance to Fusarium species. Proc Natl Acad Sci USA 110: 19324–19329 24218613 10.1073/pnas.1306373110 PMC3845197

[koac165-B65] Koch A , Biedenkopf D, Furch A, Weber L, Rossbach O, Abdellatef E, Linicus L, Johannsmeier J, Jelonek L, Goesmann A, et al (2016) An RNAi-based control of *Fusarium graminearum* infections through spraying of long dsRNAs involves a plant passage and is controlled by the fungal silencing machinery. PLoS Pathog 12: e1005901 27737019 10.1371/journal.ppat.1005901 PMC5063301

[koac165-B66] Kong L , Shi X, Chen D, Yang N, Yin C, Yang J, Wang G, Huang W, Peng H, Peng D, et al (2022) Host-induced silencing of a nematode chitin synthase gene enhances resistance of soybeans to both pathogenic *Heterodera glycines* and *Fusarium oxysporum*. Plant Biotechnol J 20: 809–811 35301818 10.1111/pbi.13808 PMC9055809

[koac165-B67] Kurata N , Kariu T, Kawano S, Kimura M (2002) Molecular cloning of cDNAs encoding ribonuclease-related proteins in *Nicotiana glutinosa* leaves, as induced in response to wounding or to TMV-infection. Biosci Biotechnol Biochem 66: 391–397 11999414 10.1271/bbb.66.391

[koac165-B68] Kuznetsov SV , Ren CC, Woodson SA, Ansari A (2008) Loop dependence of the stability and dynamics of nucleic acid hairpins. Nucleic Acids Res 36: 1098–1112 18096625 10.1093/nar/gkm1083 PMC2275088

[koac165-B69] Lalande S , Merret R, Salinas-Giegé T, Drouard L (2020) Arabidopsis tRNA-derived fragments as potential modulators of translation. RNA Biol 17: 1137–1148 31994438 10.1080/15476286.2020.1722514 PMC7549631

[koac165-B70] LeBrasseur ND , MacIntosh GC, Pérez-Amador MA, Saitoh M, Green PJ (2002) Local and systemic wound-induction of RNase and nuclease activities in Arabidopsis: RNS1 as a marker for a JA-independent systemic signaling pathway. Plant J 29: 393–403 11846873 10.1046/j.1365-313x.2002.01223.x

[koac165-B71] Lezzhov AA , Atabekova AK, Tolstyko EA, Lazareva EA, Solovyev AG (2019) RNA phloem transport mediated by pre-miRNA and viral tRNA-like structures. Plant Sci 284: 99–107 31084885 10.1016/j.plantsci.2019.04.005

[koac165-B72] Lilley CJ , Bakhetia M, Charlton WL, Urwin PE (2007) Recent progress in the development of RNA interference for plant parasitic nematodes. Mol Plant Pathol 8: 701–711 20507531 10.1111/j.1364-3703.2007.00422.x

[koac165-B73] Liu S , Ladera-Carmona MJ, Poranen MM, van Bel AJE, Kogel KH, Imani J (2021) Evaluation of dsRNA delivery methods for targeting macrophage migration inhibitory factor MIF in RNAi-based aphid control. J Plant Dis Protect 128: 1201–1212

[koac165-B74] Mamta, Reddy KR, Rajam MV (2016) Targeting chitinase gene of *Helicoverpa armigera* by host-induced RNA interference confers insect resistance in tobacco and tomato. Plant Mol Biol 90: 281–292 26659592 10.1007/s11103-015-0414-y

[koac165-B75] Maori E , Navarro IC, Boncristiani H, Seilly DJ, Rudolph KLM, Sapetschnig A, Lin CC, Ladbury JE, Evans JD, Heeney JL, et al (2019) A secreted RNA binding protein forms RNA-stabilizing granules in the honeybee royal jelly. Mol Cell 74: 598–608 e596 31051140 10.1016/j.molcel.2019.03.010 PMC6509358

[koac165-B76] Martienssen RA (2010) Heterochromatin, small RNA and post-fertilization dysgenesis in allopolyploid and interploid hybrids of Arabidopsis. New Phytol 186: 46–53 20409176 10.1111/j.1469-8137.2010.03193.x PMC3756494

[koac165-B77] Melnyk CW , Molnar A, Baulcombe DC (2011) Intercellular and systemic movement of RNA silencing signals. EMBO J 30: 3553–3563 21878996 10.1038/emboj.2011.274 PMC3181474

[koac165-B78] Movahed N , Cabanillas DG, Wan J, Vali H, Laliberté JF, Zheng H (2019) Turnip mosaic virus components are released into the extracellular space by vesicles in infected leaves. Plant Physiol 180: 1375–1388 31019004 10.1104/pp.19.00381 PMC6752911

[koac165-B79] Napoli C , Lemieux C, Jorgensen R (1990) Introduction of a chimeric *CHALCONE SYNTHASE* gene into petunia results in reversible co-suppression of homologous genes in trans. Plant Cell 2: 279–289 12354959 10.1105/tpc.2.4.279 PMC159885

[koac165-B80] Nguyen Q , Iritani A, Ohkita S, Vu BV, Yokoya K, Matsubara A, Ikeda KI, Suzuki N, Nakayashiki H (2018) A fungal Argonaute interferes with RNA interference. Nucleic Acids Res 46: 2495–2508 29309640 10.1093/nar/gkx1301 PMC5946944

[koac165-B81] Nicolas FE , Torres-Martinez S, Ruiz-Vazquez RM (2003) Two classes of small antisense RNAs in fungal RNA silencing triggered by non-integrative transgenes. EMBO J 22: 3983–3991 12881432 10.1093/emboj/cdg384 PMC169057

[koac165-B82] Notaguchi M , Wolf S, Lucas WJ (2012) Phloem-mobile Aux/IAA transcripts target to the root tip and modify root architecture. J Integr Plant Biol 54: 760–772 22925478 10.1111/j.1744-7909.2012.01155.x

[koac165-B83] Nowara D , Gay A, Lacomme C, Shaw J, Ridout C, Douchkov D, Hensel G, Kumlehn J, Schweizer P (2010) HIGS: host-induced gene silencing in the obligate biotrophic fungal pathogen *Blumeria graminis*. Plant Cell 22: 3130–3141 20884801 10.1105/tpc.110.077040 PMC2965548

[koac165-B84] Omid A , Malter D, Peleg G, Wolf S (2008) Long-distance trafficking of macromolecules in the phloem. Plant Signal Behav 3: 260–262 19704648 10.4161/psb.3.4.5196 PMC2634196

[koac165-B85] Pallas V , Gómez G (2013) Phloem RNA-binding proteins as potential components of the long-distance RNA transport system. Front Plant Sci 4: 130 23675378 10.3389/fpls.2013.00130 PMC3650515

[koac165-B86] Panwar V , McCallum B, Bakkeren G (2013) Host-induced gene silencing of wheat leaf rust fungus *Puccinia triticina* pathogenicity genes mediated by the Barley stripe mosaic virus. Plant Mol Biol 81: 595–608 23417582 10.1007/s11103-013-0022-7

[koac165-B87] Pennington HG , Jones R, Kwon S, Bonciani G, Thieron H, Chandler T, Luong P, Morgan SN, Przydacz M, Bozkurt T, et al (2019) The fungal ribonuclease-like effector protein CSEP0064/BEC1054 represses plant immunity and interferes with degradation of host ribosomal RNA. PLoS Pathog 15: e1007620 30856238 10.1371/journal.ppat.1007620 PMC6464244

[koac165-B88] Pitino M , Coleman AD, Maffei ME, Ridout CJ, Hogenhout SA (2011) Silencing of aphid genes by dsRNA feeding from plants. PLoS One 6: e25709 21998682 10.1371/journal.pone.0025709 PMC3187792

[koac165-B89] Pliego C , Nowara D, Bonciani G, Gheorghe DM, Xu R, Surana P, Whigham E, Nettleton D, Bogdanove AJ, Wise RP, et al (2013) Host-induced gene silencing in barley powdery mildew reveals a class of ribonuclease-like effectors. Mol Plant Microbe Interact 26: 633–642 23441578 10.1094/MPMI-01-13-0005-R

[koac165-B90] Portela A , Esteller M (2010) Epigenetic modifications and human disease. Nat Biotechnol 28: 1057–1068 20944598 10.1038/nbt.1685

[koac165-B91] Potrokhov A , Sosnovska D, Ovcharenko O, Budzanivska I, Rudas V, Kuchuk M (2021) Increased ribonuclease activity in *Solanum tuberosum* L. transformed with heterologous genes of apoplastic ribonucleases as a putative approach for production of virus resistant plants. Turk J Biol 45: 79–87 33597824 10.3906/biy-2007-87 PMC7877712

[koac165-B92] Qi T , Zhu X, Tan C, Liu P, Guo J, Kang Z, Guo J (2018) Host-induced gene silencing of an important pathogenicity factor PsCPK1 in *Puccinia striiformis* f. sp. tritici enhances resistance of wheat to stripe rust. Plant Biotechnol J 16: 797–807 28881438 10.1111/pbi.12829 PMC5814584

[koac165-B93] Qiao L , Lan C, Capriotti L, Ah-Fong A, Nino Sanchez J, Hamby R, Heller J, Zhao H, Glass NL, Judelson HS, et al (2021) Spray-induced gene silencing for disease control is dependent on the efficiency of pathogen RNA uptake. Plant Biotechnol J 19: 1756–1768 33774895 10.1111/pbi.13589 PMC8428832

[koac165-B94] Raruang Y , Omolehin O, Hu D, Wei Q, Han ZQ, Rajasekaran K, Cary JW, Wang K, Chen ZY (2020) Host induced gene silencing targeting *Aspergillus flavus* aflM reduced aflatoxin contamination in transgenic maize under field conditions. Front Microbiol 11: 754 32411110 10.3389/fmicb.2020.00754 PMC7201132

[koac165-B95] Rodríguez-Celma J , Ceballos-Laita L, Grusak MA, Abadía J, López-Millán AF (2016) Plant fluid proteomics: delving into the xylem sap, phloem sap and apoplastic fluid proteomes. Biochim Biophys Acta 1864: 991–1002 27033031 10.1016/j.bbapap.2016.03.014

[koac165-B96] Romano N , Macino G (1992) Quelling: transient inactivation of gene expression in *Neurospora crassa* by transformation with homologous sequences. Mol Microbiol 6: 3343–3353 1484489 10.1111/j.1365-2958.1992.tb02202.x

[koac165-B97] Rountree MR , Selker EU (1997) DNA methylation inhibits elongation but not initiation of transcription in *Neurospora crassa*. Genes Dev 11: 2383–2395 9308966 10.1101/gad.11.18.2383 PMC316521

[koac165-B98] Ruiz-Medrano R , Xoconostle-Cázares B, Lucas WJ (1999) Phloem long-distance transport of CmNACP mRNA: implications for supracellular regulation in plants. Development 126: 4405–4419 10498677 10.1242/dev.126.20.4405

[koac165-B99] Rutter BD , Innes RW (2017) Extracellular vesicles isolated from the leaf apoplast carry stress-response proteins. Plant Physiol 173: 728–741 27837092 10.1104/pp.16.01253 PMC5210723

[koac165-B100] Sambade A , Brandner K, Hofmann C, Seemanpillai M, Mutterer J, Heinlein M (2008) Transport of TMV movement protein particles associated with the targeting of RNA to plasmodesmata. Traffic 9: 2073–2088 19281527 10.1111/j.1600-0854.2008.00824.x

[koac165-B101] San Miguel K , Scott JG (2016) The next generation of insecticides: dsRNA is stable as a foliar-applied insecticide. Pest Manag Sci 72: 801–809 26097110 10.1002/ps.4056

[koac165-B102] Sangaev SS , Trifonova EA, Titov SE, Romanova AV, Kolodiazhnaia Ia S, Komarova ML, Sapotskiĭ MV, Malinovskiĭ VI, Kochetov AV, Shumnyĭ VK (2007) Effective expression of the gene encoding an extracellular ribonuclease of *Zinnia elegans* in the SR1 *Nicotiana tabacum* plants. Genetika 43: 1002–1005 17899821

[koac165-B103] Sasaki T , Chino M, Hayashi H, Fujiwara T (1998) Detection of several mRNA species in rice phloem sap. Plant Cell Physiol 39: 895–897 9787465 10.1093/oxfordjournals.pcp.a029451

[koac165-B104] Secic E , Zanini S, Kogel KH (2019) Further elucidation of the Argonaute and Dicer protein families in the model grass species *Brachypodium distachyon*. Front Plant Sci 10: 1332 31708948 10.3389/fpls.2019.01332 PMC6822278

[koac165-B105] Senthil-Kumar M , Mysore KS (2011) Virus-induced gene silencing can persist for more than 2 years and also be transmitted to progeny seedlings in *Nicotiana benthamian*a and tomato. Plant Biotechnol J 9: 797–806 21265998 10.1111/j.1467-7652.2011.00589.x

[koac165-B106] Serra MJ , Lyttle MH, Axenson TJ, Schadt CA, Turner DH (1993) RNA hairpin loop stability depends on closing base pair. Nucleic Acids Res 21: 3845–3849 7690127 10.1093/nar/21.16.3845 PMC309905

[koac165-B107] Shahid S , Kim G, Johnson NR, Wafula E, Wang F, Coruh C, Bernal-Galeano V, Phifer T, dePamphilis CW, Westwood JH, et al (2018) MicroRNAs from the parasitic plant *Cuscuta campestris* target host messenger RNAs. Nature 553: 82–85 29300014 10.1038/nature25027

[koac165-B108] Sijen T , Fleenor J, Simmer F, Thijssen KL, Parrish S, Timmons L, Plasterk RH, Fire A (2001) On the role of RNA amplification in dsRNA-triggered gene silencing. Cell 107: 465–476 11719187 10.1016/s0092-8674(01)00576-1

[koac165-B109] Singh S , Gupta M, Pandher S, Kaur G, Rathore P, Palli SR (2018) Selection of housekeeping genes and demonstration of RNAi in cotton leafhopper, *Amrasca biguttula biguttula* (Ishida). PLoS One 13: e0191116 29329327 10.1371/journal.pone.0191116 PMC5766320

[koac165-B110] Smith NA , Singh SP, Wang MB, Stoutjesdijk PA, Green AG, Waterhouse PM (2000) Total silencing by intron-spliced hairpin RNAs. Nature 407: 319–320 11014180 10.1038/35030305

[koac165-B111] Song XS , Gu KX, Duan XX, Xiao XM, Hou YP, Duan YB, Wang JX, Yu N, Zhou MG (2018) Secondary amplification of siRNA machinery limits the application of spray-induced gene silencing. Mol Plant Pathol 19: 2543–2560 30027625 10.1111/mpp.12728 PMC6638038

[koac165-B112] Sugawara T , Trifonova EA, Kochetov AV, Kanayama Y (2016) Expression of an extracellular ribonuclease gene increases resistance to Cucumber mosaic virus in tobacco. BMC Plant Biol 16: 246 28105959 10.1186/s12870-016-0928-8 PMC5123310

[koac165-B113] Taochy C , Gursanscky NR, Cao J, Fletcher SJ, Dressel U, Mitter N, Tucker MR, Koltunow AMG, Bowman JL, Vaucheret H, et al (2017) A genetic screen for impaired systemic RNAi highlights the crucial role of DICER-LIKE 2. Plant Physiol 175: 1424–1437 28928141 10.1104/pp.17.01181 PMC5664484

[koac165-B114] Thieme CJ , Rojas-Triana M, Stecyk E, Schudoma C, Zhang W, Yang L, Miñambres M, Walther D, Schulze WX, Paz-Ares J, et al (2015) Endogenous Arabidopsis messenger RNAs transported to distant tissues. Nat Plants 1: 15025 27247031 10.1038/nplants.2015.25

[koac165-B115] Tian B , Li J, Oakley TR, Todd TC, Trick HN (2016) Host-derived artificial microRNA as an alternative method to improve soybean resistance to soybean cyst nematode. Genes (Basel) 7: 122 27941644 10.3390/genes7120122 PMC5192498

[koac165-B116] Tosar JP , Cayota A (2020) Extracellular tRNAs and tRNA-derived fragments. RNA Biol 17: 1149–1167 32070197 10.1080/15476286.2020.1729584 PMC7549618

[koac165-B117] Urwin PE , Lilley CJ, Atkinson HJ (2002) Ingestion of double-stranded RNA by preparasitic juvenile cyst nematodes leads to RNA interference. Mol Plant Microbe Interact 15: 747–752 12182331 10.1094/MPMI.2002.15.8.747

[koac165-B118] Vecenie CJ , Serra MJ (2004) Stability of RNA hairpin loops closed by AU base pairs. Biochemistry 43: 11813–11817 15362866 10.1021/bi049954i

[koac165-B119] Vetukuri RR , Avrova AO, Grenville-Briggs LJ, Van West P, Soderbom F, Savenkov EI, Whisson SC, Dixelius C (2011) Evidence for involvement of Dicer-like, Argonaute and histone deacetylase proteins in gene silencing in *Phytophthora infestans*. Mol Plant Pathol 12: 772–785 21726377 10.1111/j.1364-3703.2011.00710.x PMC6640358

[koac165-B120] Villarroya-Beltri C , Gutiérrez-Vázquez C, Sánchez-Cabo F, Pérez-Hernández D, Vázquez J, Martin-Cofreces N, Martinez-Herrera DJ, Pascual-Montano A, Mittelbrunn M, Sánchez-Madrid F (2013) Sumoylated hnRNPA2B1 controls the sorting of miRNAs into exosomes through binding to specific motifs. Nat Commun 4: 2980 24356509 10.1038/ncomms3980 PMC3905700

[koac165-B121] Wan J , Laliberté JF (2015) Membrane-associated virus replication complexes locate to plant conducting tubes. Plant Signal Behav 10: e1042639 25955489 10.1080/15592324.2015.1042639 PMC4622829

[koac165-B122] Wang M , Weiberg A, Lin FM, Thomma BP, Huang HD, Jin H (2016) Bidirectional cross-kingdom RNAi and fungal uptake of external RNAs confer plant protection. Nat Plants 2: 16151 27643635 10.1038/nplants.2016.151 PMC5040644

[koac165-B123] Wang MB , Waterhouse PM (2000) High-efficiency silencing of a beta-glucuronidase gene in rice is correlated with repetitive transgene structure but is independent of DNA methylation. Plant Mol Biol 43: 67–82 10949375 10.1023/a:1006490331303

[koac165-B124] Wang MB , Helliwell CA, Wu LM, Waterhouse PM, Peacock WJ, Dennis ES (2008) Hairpin RNAs derived from RNA polymerase II and polymerase III promoter-directed transgenes are processed differently in plants. RNA 14: 903–913 18367720 10.1261/rna.760908 PMC2327362

[koac165-B125] Waterhouse PM , Helliwell CA (2003) Exploring plant genomes by RNA-induced gene silencing. Nat Rev Genet 4: 29–38 12509751 10.1038/nrg982

[koac165-B126] Waterhouse PM , Graham MW, Wang MB (1998) Virus resistance and gene silencing in plants can be induced by simultaneous expression of sense and antisense RNA. Proc Natl Acad Sci USA 95: 13959–13964 9811908 10.1073/pnas.95.23.13959 PMC24986

[koac165-B127] Weiberg A , Wang M, Lin FM, Zhao H, Zhang Z, Kaloshian I, Huang HD, Jin H (2013) Fungal small RNAs suppress plant immunity by hijacking host RNA interference pathways. Science 342: 118–123 24092744 10.1126/science.1239705 PMC4096153

[koac165-B128] Werner BT , Gaffar FY, Schuemann J, Biedenkopf D, Koch AM (2020) RNA-spray-mediated silencing of *Fusarium graminearum* AGO and DCL genes improve barley disease resistance. Front Plant Sci 11: 476 32411160 10.3389/fpls.2020.00476 PMC7202221

[koac165-B129] Wesley SV , Helliwell CA, Smith NA, Wang MB, Rouse DT, Liu Q, Gooding PS, Singh SP, Abbott D, Stoutjesdijk PA, et al (2001) Construct design for efficient, effective and high-throughput gene silencing in plants. Plant J 27: 581–590 11576441 10.1046/j.1365-313x.2001.01105.x

[koac165-B130] Wille AC , Lucas WJ (1984) Ultrastructural and histochemical studies on guard cells. Planta 160: 129–142 24258415 10.1007/BF00392861

[koac165-B131] Williams L , Grigg SP, Xie M, Christensen S, Fletcher JC (2005) Regulation of Arabidopsis shoot apical meristem and lateral organ formation by microRNA miR166g and its AtHD-ZIP target genes. Development 132: 3657–3668 16033795 10.1242/dev.01942

[koac165-B132] Winston WM , Molodowitch C, Hunter CP (2002) Systemic RNAi in *C. elegans* requires the putative transmembrane protein SID-1. Science 295: 2456–2459 11834782 10.1126/science.1068836

[koac165-B133] Winter J , Link S, Witzigmann D, Hildenbrand C, Previti C, Diederichs S (2013) Loop-miRs: active microRNAs generated from single-stranded loop regions. Nucleic Acids Res 41: 5503–5512 23580554 10.1093/nar/gkt251 PMC3664828

[koac165-B134] Wu H , Li B, Iwakawa HO, Pan Y, Tang X, Ling-Hu Q, Liu Y, Sheng S, Feng L, Zhang H, et al (2020) Plant 22-nt siRNAs mediate translational repression and stress adaptation. Nature 581: 89–93 32376953 10.1038/s41586-020-2231-y

[koac165-B135] Wu L , Zhang Q, Zhou H, Ni F, Wu X, Qi Y (2009) Rice MicroRNA effector complexes and targets. Plant Cell 21: 3421–3435 19903869 10.1105/tpc.109.070938 PMC2798332

[koac165-B136] Xiao D , Gao X, Xu J, Liang X, Li Q, Yao J, Zhu KY (2015) Clathrin-dependent endocytosis plays a predominant role in cellular uptake of double-stranded RNA in the red flour beetle. Insect Biochem Mol Biol 60: 68–77 25863352 10.1016/j.ibmb.2015.03.009

[koac165-B137] Xoconostle-Cázares B , Xiang Y, Ruiz-Medrano R, Wang HL, Monzer J, Yoo BC, McFarland KC, Franceschi VR, Lucas WJ (1999) Plant paralog to viral movement protein that potentiates transport of mRNA into the phloem. Science 283: 94–98 9872750 10.1126/science.283.5398.94

[koac165-B138] Xu HJ , Chen T, Ma XF, Xue J, Pan PL, Zhang XC, Cheng JA, Zhang CX (2013) Genome-wide screening for components of small interfering RNA (siRNA) and micro-RNA (miRNA) pathways in the brown planthopper, *Nilaparvata lugens* (Hemiptera: Delphacidae). Insect Mol Biol 22: 635–647 23937246 10.1111/imb.12051

[koac165-B139] Xu P , Zhang Y, Kang L, Roossinck MJ, Mysore KS (2006) Computational estimation and experimental verification of off-target silencing during posttranscriptional gene silencing in plants. Plant Physiol 142: 429–440 16920874 10.1104/pp.106.083295 PMC1586062

[koac165-B140] Xu W , Han Z (2008) Cloning and phylogenetic analysis of *sid-1*-like genes from aphids. J Insect Sci 8: 1–6 10.1673/031.008.3001 PMC3061602 20302524

[koac165-B141] Yan Y , Ham BK, Chong YH, Yeh SD, Lucas WJ (2020) A plant SMALL RNA-BINDING PROTEIN 1 family mediates cell-to-cell trafficking of RNAi signals. Mol Plant 13: 321–335 31812689 10.1016/j.molp.2019.12.001

[koac165-B142] Yoo BC , Kragler F, Varkonyi-Gasic E, Haywood V, Archer-Evans S, Lee YM, Lough TJ, Lucas WJ (2004) A systemic small RNA signaling system in plants. Plant Cell 16: 1979–2000 15258266 10.1105/tpc.104.023614 PMC519190

[koac165-B143] Yue SB , Trujillo RD, Tang Y, O'Gorman WE, Chen CZ (2011) Loop nucleotides control primary and mature miRNA function in target recognition and repression. RNA Biol 8: 1115–1123 22142974 10.4161/rna.8.6.17626 PMC3256424

[koac165-B144] Zand Karimi H , Baldrich P, Rutter BD, Borniego L, Zajt KK, Meyers BC, Innes RW (2022) Arabidopsis apoplastic fluid contains sRNA- and circular RNA-protein complexes that are located outside extracellular vesicles. Plant Cell 34: 1863–1881 35171271 10.1093/plcell/koac043 PMC9048913

[koac165-B145] Zhang H , Goh NS, Wang JW, Pinals RL, González-Grandío E, Demirer GS, Butrus S, Fakra SC, Del Rio Flores A, Zhai R, et al (2022) Nanoparticle cellular internalization is not required for RNA delivery to mature plant leaves. Nat Nanotechnol 17: 197–205 34811553 10.1038/s41565-021-01018-8 PMC10519342

[koac165-B146] Zhang H , Xia R, Meyers BC, Walbot V (2015a) Evolution, functions, and mysteries of plant ARGONAUTE proteins. Curr Opin Plant Biol 27: 84–90 26190741 10.1016/j.pbi.2015.06.011

[koac165-B147] Zhang J , Khan SA, Hasse C, Ruf S, Heckel DG, Bock R (2015b) Pest control. Full crop protection from an insect pest by expression of long double-stranded RNAs in plastids. Science 347: 991–994 25722411 10.1126/science.1261680

[koac165-B148] Zhang L , Tian LH, Zhao JF, Song Y, Zhang CJ, Guo Y (2009a) Identification of an apoplastic protein involved in the initial phase of salt stress response in rice root by two-dimensional electrophoresis. Plant Physiol 149: 916–928 19036832 10.1104/pp.108.131144 PMC2633861

[koac165-B149] Zhang S , Sun L, Kragler F (2009b) The phloem-delivered RNA pool contains small noncoding RNAs and interferes with translation. Plant Physiol 150: 378–387 19261735 10.1104/pp.108.134767 PMC2675743

[koac165-B150] Zhang T , Zhao YL, Zhao JH, Wang S, Jin Y, Chen ZQ, Fang YY, Hua CL, Ding SW, Guo HS (2016a) Cotton plants export microRNAs to inhibit virulence gene expression in a fungal pathogen. Nat Plants 2: 16153 27668926 10.1038/nplants.2016.153

[koac165-B151] Zhang X , Zhao H, Gao S, Wang WC, Katiyar-Agarwal S, Huang HD, Raikhel N, Jin H (2011) Arabidopsis Argonaute 2 regulates innate immunity via miRNA393 ()-mediated silencing of a Golgi-localized SNARE gene, MEMB12. Mol Cell 42: 356–366 21549312 10.1016/j.molcel.2011.04.010 PMC3101262

[koac165-B152] Zhang Z , Liu X, Guo X, Wang XJ, Zhang X (2016b) Arabidopsis AGO3 predominantly recruits 24-nt small RNAs to regulate epigenetic silencing. Nat Plants 2: 16049 27243648 10.1038/nplants.2016.49

[koac165-B153] Zhao D , Zhong GY, Song GQ (2020) Transfer of endogenous small RNAs between branches of scions and rootstocks in grafted sweet cherry trees. PLoS One 15: e0236376 32722723 10.1371/journal.pone.0236376 PMC7386610

[koac165-B154] Zhou X , Wang L, Zou W, Chen X, Roizman B, Zhou GG (2020) hnRNPA2B1 associated with recruitment of RNA into exosomes pays a key role in Herpes Simplex Virus 1 release from infected cells. J Virol 94: e00367-20 32295924 10.1128/JVI.00367-20 PMC7307161

[koac165-B155] Zhu L , Zhu J, Liu Z, Wang Z, Zhou C, Wang H (2017) Host-induced gene silencing of rice blast fungus *Magnaporthe oryzae* pathogenicity genes mediated by the brome mosaic virus. Genes (Basel) 8: 241 28954400 10.3390/genes8100241 PMC5664091

[koac165-B156] Zilberman D , Cao X, Johansen LK, Xie Z, Carrington JC, Jacobsen SE (2004) Role of Arabidopsis ARGONAUTE4 in RNA-directed DNA methylation triggered by inverted repeats. Curr Biol 14: 1214–1220 15242620 10.1016/j.cub.2004.06.055

